# Transcriptome analysis of *Clinopodium gracile* (Benth.) Matsum and identification of genes related to Triterpenoid Saponin biosynthesis

**DOI:** 10.1186/s12864-020-6454-y

**Published:** 2020-01-15

**Authors:** Chunmiao Shan, Chenkai Wang, Shengxiang Zhang, Yuanyuan Shi, Kelong Ma, Qingshan Yang, Jiawen Wu

**Affiliations:** 1 Anhui University of Chinese Medicine and Anhui Academy of Chinese Medicine, Hefei, 230038 China; 2 Key Laboratory of Xin’an Medicine, Ministry of Education, Anhui University of Chinese Medicine, Hefei, 230038 China; 3 Clinical College of Integrated Traditional Chinese and Western Medicine, Anhui University of Chinese Medicine, Hefei, 230012 China; 4 Synergetic Innovation Center of Anhui Authentic Chinese Medicine Quality Improvement, Hefei, 230012 China

**Keywords:** *Clinopodium gracile* (Benth.) Matsum, Transcriptome, RNA-Seq, Triterpenoid saponin biosynthesis, Differentially expressed genes

## Abstract

**Background:**

*Clinopodium gracile* (Benth.) Matsum (*C. gracile*) is an annual herb with pharmacological properties effective in the treatment of various diseases, including hepatic carcinoma. Triterpenoid saponins are crucial bioactive compounds in *C. gracile*. However, the molecular understanding of the triterpenoid saponin biosynthesis pathway remains unclear.

**Results:**

In this study, we performed RNA sequencing (RNA-Seq) analysis of the flowers, leaves, roots, and stems of *C. gracile* plants using the BGISEQ-500 platform. The assembly of transcripts from all four types of tissues generated 128,856 unigenes, of which 99,020 were mapped to several public databases for functional annotation. Differentially expressed genes (DEGs) were identified via the comparison of gene expression levels between leaves and other tissues (flowers, roots, and stems). Multiple genes encoding pivotal enzymes, such as squalene synthase (SS), or transcription factors (TFs) related to triterpenoid saponin biosynthesis were identified and further analyzed. The expression levels of unigenes encoding important enzymes were verified by quantitative real-time PCR (qRT-PCR). Different chemical constituents of triterpenoid saponins were identified by Ultra-Performance Liquid Chromatography coupled with quadrupole time-of-flight mass spectrometry (UPLC/Q-TOF-MS).

**Conclusions:**

Our results greatly extend the public transcriptome dataset of *C. gracile* and provide valuable information for the identification of candidate genes involved in the biosynthesis of triterpenoid saponins and other important secondary metabolites.

## Background

*Clinopodium gracile* (Benth.) Matsum (*C. gracile*), known as the tower flower, is a traditional Chinese herb, which belongs to Lamiaceae and grows on wasteland, roadsides, and hillsides [1, 2]. Approximately 20 *Clinopodium* species are found in Europe, Central Asia, and East Asia [3]. According to the results of previous studies, triterpenoid saponins in *C. gracile* exhibit several pharmacological effects, as these possess anti-inflammatory [4], anti-hepatoma [4, 5], cardioprotective [6], anti-tumor [7], and immunoregulatory [8] properties. Triterpenoid saponins were synthesized from two C5 isoprene units, isopentenyl pyrophosphate (IPP) and dimethylallyl diphosphate (DMAPP). These components were condensed in a sequential manner by prenyltransferases, resulting in the formation of prenyl diphosphates, such as geranyl pyrophosphate (GPP) and farnesyl pyrophosphate (FPP), which are further transformed into the carbocyclic skeleton of triterpenoid saponins by the action of squalene synthase (SS) and squalene epoxidase (SE). Finally, the backbone is chemically modified by cytochrome P450 monooxygenase (CYP450) and UDP-glycosyltransferase (UGT), resulting in the production of different types of triterpenoid saponins [9–12]. However, genes encoding key triterpenoid saponin biosynthesis enzymes in *C. gracile* are largely unknown.

Squalene synthase (SS; EC 2.5.1.21) is a key bifunctional enzyme in the terpenoid biosynthesis pathway; SS first catalyzes the formation of presqualene diphosphate (PSPP) from two molecules of FPP and then converts PSPP to squalene with NADPH and Mg^2+^ ions [13, 14]. The level of *SS* gene expression shows a positive correlation with the amount of triterpenes [15]. Although *SS* cDNAs have been cloned and analyzed from a number of herbal plant species, such as *Glycyrrhiza glabra* [16], *Siraitia grosvenorii* [17], and *Lotus japonicas* [18], no sequence or structural information is available on the *SS* gene in *C. gracile*.

RNA sequencing (RNA-Seq) analysis has been used to capture both coding and non-coding sequences and to quantify gene expression not only in a heterogeneous mixture of cells, tissues, and organs but also at the whole organism level [19]. Thus, RNA-Seq is a powerful tool for acquiring gene expression information and is of great significance in the mining of functional genes, analysis of gene expression profiles, and discovery of genetic metabolic networks [20]. At present, transcriptome sequencing has been applied to medicinal plants of the Labiatae family, including *Scutellaria baicalensis* Georgi [21], *Ocimum sanctum* and *Ocimum basilicum* [22], *Mentha piperita* and *Mentha arvensis* [23], and *Clinopodium chinense* [24]; however, the use of RNA-Seq analysis has not been reported in *C. gracile* to date.

In this study, we performed deep transcriptome analysis of four different tissues (flowers, leaves, roots, and stems) of *C. gracile* plants and identified genes potentially involved in triterpenoid saponin biosynthesis. This work lays a foundation for further exploration of the molecular mechanism of triterpenoid saponin biosynthesis in *C. gracile*.

## Results

### Total saponin content in different tissues of *C. gracile*

**Fig. 1:**
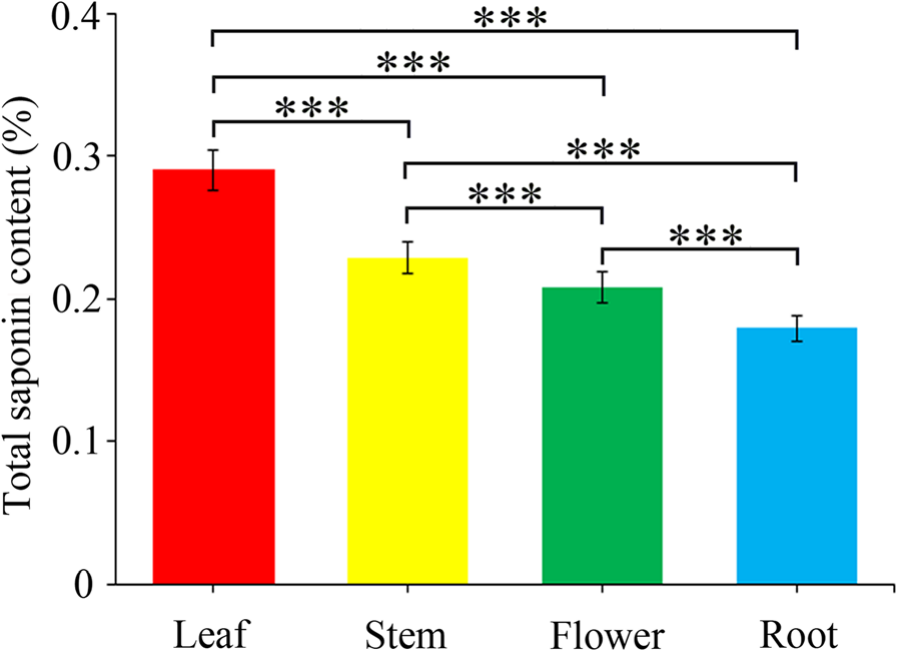
The total saponin content of leaves, stems, flowers, and roots of *Clinopodium gracile* (Benth.) Matsum plants. The X-axis indicates the type of *C. gracile* tissues, and the Y-axis indicates the total saponin content. Significant differences were determined by analysis of variance (ANOVA)

Saponins were extracted from approximately 0.1 g dried powder of flowers, leaves, stems, and roots of *C. gracile*. Total saponin content was the highest in leaves (0.29%), followed by stems (0.23%), flowers (0.21%), and roots (0.18%), with a standard error of 0.0005, 0.0004, 0.0033, and 0.0011, respectively. The results of analysis of variance (ANOVA) of *C. gracile* data showed that the differences among the total saponin content of the four tissues were statistically significant (Fig. 1, Additional file 1: Table S1).

### Analysis of triterpenoid saponins in *C. gracile* by UPLC/Q-TOF-MS

**Fig. 2:**
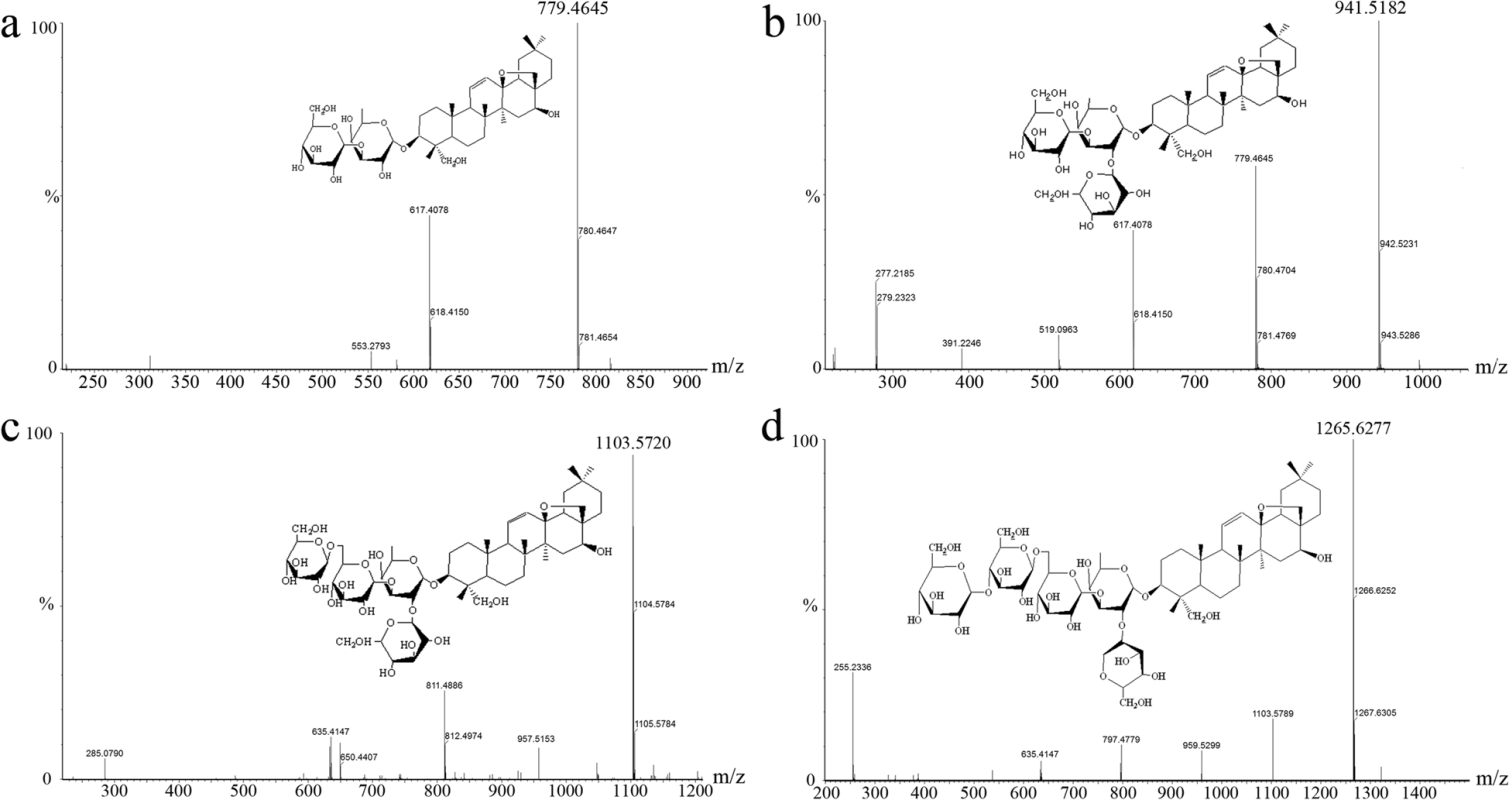
MS/MS spectra of Saikosaponin a (**a**), Buddlejasaponin IV (**b**), Clinoposaponin III (**c**) and Clinoposaponin V (**d**) of *C. gracile* by UPLC/Q-TOF-MS in negative ion mode

Qualitative analysis of triterpenoid saponin metabolites of *C. gracile* was conducted by UPLC/Q-TOF-MS. The results confirmed the presence of Buddlejasaponin IV in *C. gracile*. Additionally, Saikosaponin a, Clinoposaponin III, and Clinoposaponin V probably also exist in *C. gracile*, according to the retention time (t_R_), maximum ultraviolet absorption wavelength (λmax), molecular ion peak, and ESI-MS data (Fig. 2, Additional file 2: Table S2).

### RNA sequencing and the de novo assembly of *C. gracile* transcriptome

RNA-Seq analysis of *C. gracile* flowers, leaves, stems, and roots generated 43.81 Gb of high quality reads. Full-length transcripts were reconstructed, and a total of 128,856 unigenes, which refer to a uniquely assembled transcript or a cluster of genes that perform a particular function, were generated using Trinity and TGICL, with a mean length of 1354 bp and an N50 value of 2268 bp. Of the 128,856 unigenes, 47.37% (61,043 unigenes) were longer than 1000 bp, and 34.87% (44,929 unigenes) were longer than 1500 bp (Additional file 3: Figure S1).

### Functional classification and expressed overview of unigenes

Of the 128,856 unigenes, 38.99, 57.16, 58.94, 72.57, 54.23, 54.96 and 55.67% mapped to GO, KEGG, KOG, NR, NT, Pfam, and SwissProt databases, respectively. Additionally, 99,020 (76.85%) unigenes were annotated in at least one public database, while a total of 25,975 (20.16%) unigenes were co-annotated in five databases (Table 1). Species distribution analysis showed that *C. gracile* unigenes showed the highest homology to *Sesamum indicum* sequences (40,961 unigenes; 43.81%), followed by *Erythranthe guttata* (14,690 unigenes; 15.71%) and *Daucus carota subsp. sativus* (8866 unigenes; 9.48%) (Additional file 4: Figure S2).

**Table 1:** *Clinopodium gracile* unigenes annotated using seven databases

Database	Unigene number	Percentage of unigene annotation (%)
NR	93,515	72.57
NT	69,883	54.23
Swissprot	71,728	55.67
KEGG	73,648	57.16
KOG	75,952	58.94
Pfam	70,814	54.96
GO	50,235	38.99
Intersection	25,975	20.16
Overall	99,020	76.85

The GO database divided 57.72% of unigenes into “cellular component”, 79.93% into “molecular function”, and 49.12% into “biological process”, and 50,235 unigenes were categorized into at least one GO term. Furthermore, “membrane part” (15,180 unigenes [43.72%]) and “cell” (12,907 unigenes [37.18%]) were the most enriched GO terms within the “cellular component” category; “catalytic activity” (24,182 unigenes [43.45%]), and “binding” (24,147 unigenes [43.48%]) were the most enriched GO terms under “molecular function”; “cellular process” (13,531 unigenes [43.45%]), and “biological regulation” (4852 unigenes [15.58%]) were the most enriched GO terms under “biological process” (Additional file 5: Figure S3).

**Fig. 3:**
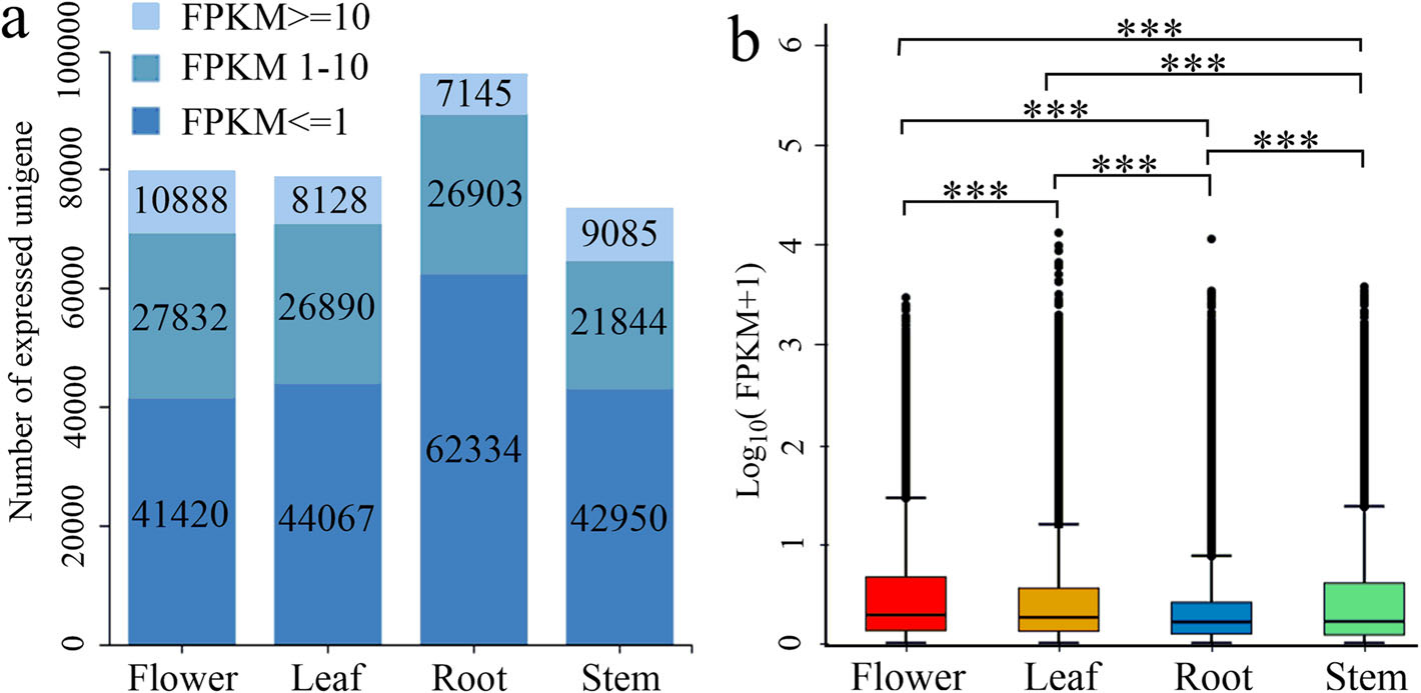
Gene expression profiles in the four tissues of *C. gracile* plants. (a) unigenes expression distribution in all four tissues (leaves, flowers, stems, and roots). The X-axis represents the sample name, and Y-axis represents the transcript levels. The level of gene expression is indicated by varying color intensity: low expression (FPKM ≤1), medium expression (FPKM 1–10), and high expression (FPKM ≥10). (b) Expressed unigenes in the four tissues by a boxplot. The X-axis indicates the type of *C. gracile* tissues and the Y-axis indicates the log10 (FPKM + 1) values of unigenes. Significant differences were determined by the Kruskal–Wallis nonparametric test

A total of 80,140, 79,085, 96,382, and 73,919 unigenes were counted in the RNA-Seq data sets of flower, leaf, stem, and root tissues, of which 10,888, 8128, 7145 and 9085 unigenes with FPKM ≥10 showed high expression, 27,832, 26,890, 26,903 and 21,844 unigenes with FPKM = 1–10 showed medium expression, and 41,420, 44,067, 62,334 and 42,950 unigenes with FPKM ≤1 showed low expression, respectively (Fig. 3a) [25, 26]. The overall expression level of unigenes was the highest in flowers, followed by stems, leaves, and roots (Fig. 3b).

### Identification of candidate genes involved in the triterpenoid biosynthesis pathway

**Fig. 4:**
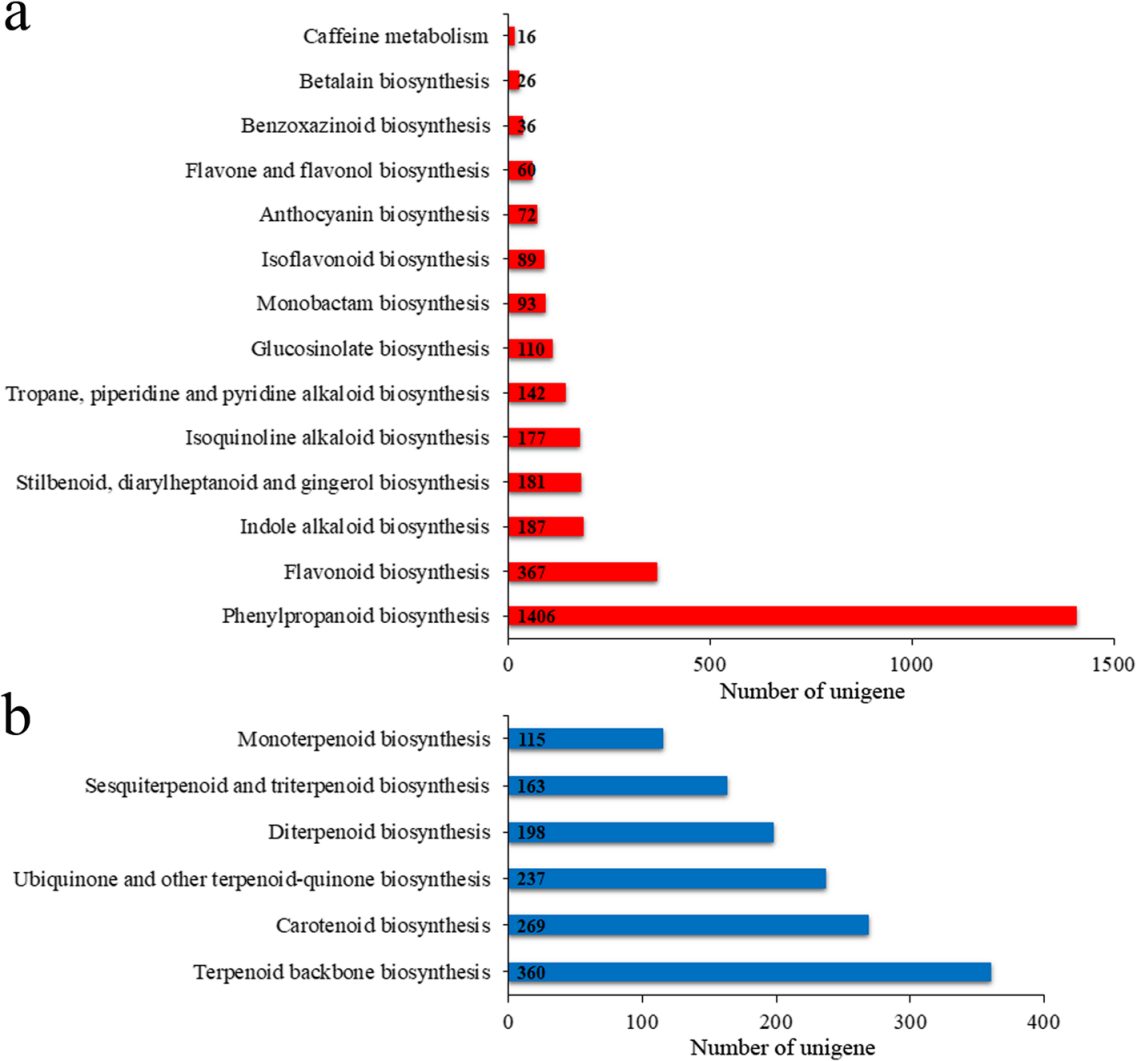
KEGG annotation of *C. gracile* unigenes (**a** and **b**) The number of unigenes involved in the biosynthesis of other secondary metabolites (**a**) and terpenoids (**b**)

Gene function annotation using the KEGG database assigned 73,648 unigenes to 136 pathways (20 subcategories), 14 of which were related to the biosynthesis of secondary metabolites (Additional file 6: Figure S4 and Additional file 7: Table S3). A total of 1406 unigenes were enriched in the phenylpropanoid biosynthesis pathway (Fig. 4a). Terpenoid metabolism involves six pathways; the largest number of unigenes (360) mapped to “Terpenoid backbone biosynthesis,” followed by “Carotenoid biosynthesis,” “Ubiquinone and other terpenoid quinone biosynthesis,” “Diterpenoid biosynthesis,” and “Sesquiterpenoid and triterpenoid biosynthesis” (Fig. 4b).

**Fig. 5:**
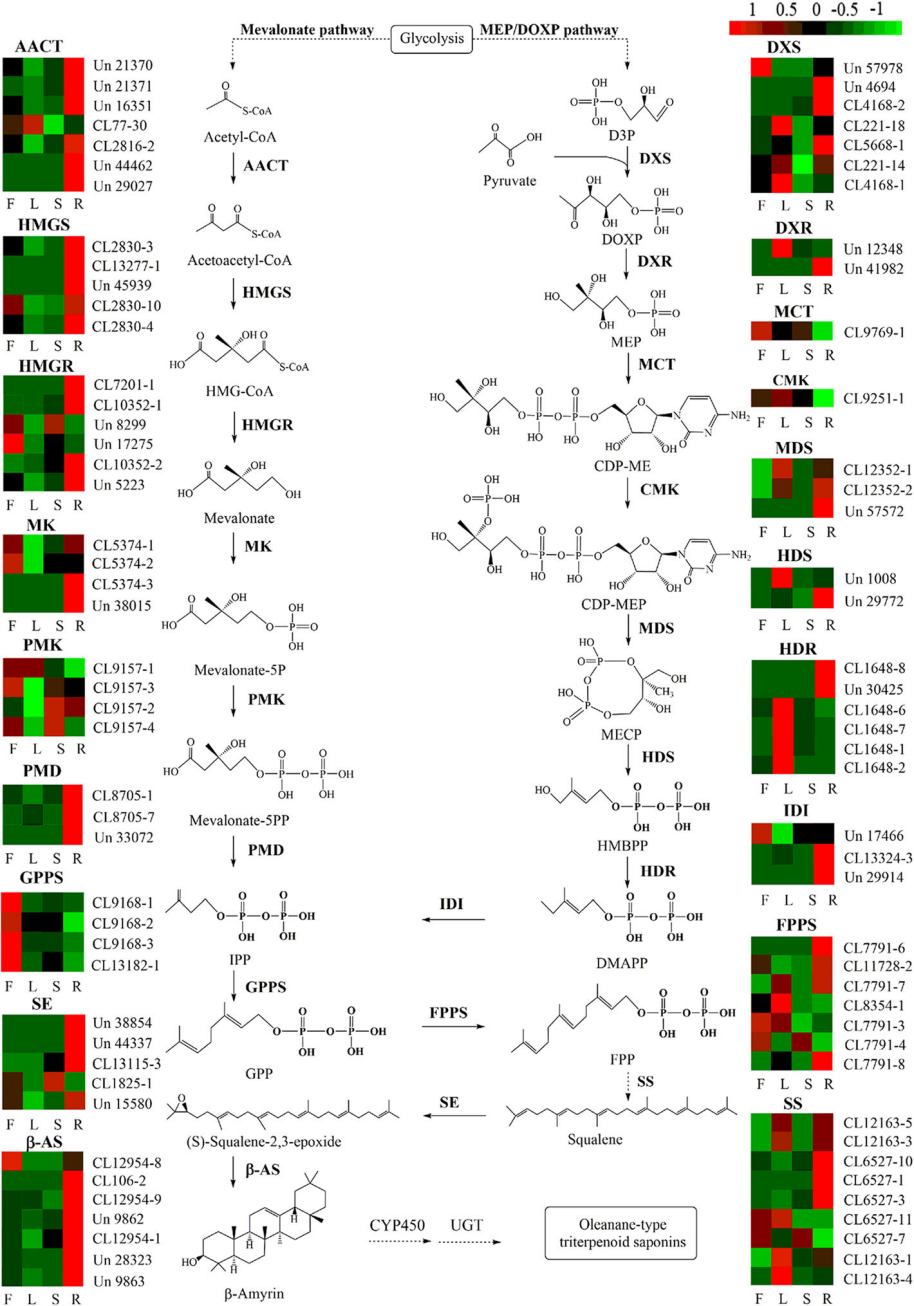
Proposed pathways for triterpenoid saponin biosynthesis in *C. gracile.* Expression levels of unigenes encoding enzymes that catalyze each step of the triterpenoid saponin biosynthesis pathway are shown. “CL” and “Un” indicates a cluster of transcripts and unigenes, respectively. F, flowers; L, leaves; S, stems; R, roots. The higher and lower expression level of unigenes are indicated in red and green, respectively

A total of 523 unigenes were enriched in the “terpenoid backbone biosynthesis” (ko00900) and “sesquiterpenoid and triterpenoid biosynthesis” (ko00909) pathways. Additionally, 281 unigenes encoded key enzymes involved in these pathways, including 3-hydroxy-3-methylglutaryl coenzyme A reductase (HMGR; 17 unigenes), 1-deoxy-D-xylulose-5-phosphate synthase (DXS; 36 unigenes), 1-deoxy-D-xylulose-5-phosphate reductase (DXR; 2 unigenes), 4-hydroxy-3-methylbut-2-en-1-yl diphosphate reductase (HDR; 11 unigenes), isopentenyl-diphosphate Delta-isomerase (IDI; 12 unigenes), SS (19 unigenes), squalene epoxidase (SE, 14 unigenes), and beta-amyrin synthase (β-AS; 32 unigenes). A total of 108 unigenes encoded 6 key enzymes are involved in the MVA pathway; 74 unigenes encoded 8 key enzymes of the MEP pathway; and 99 unigenes encoded 5 key enzymes involved in the conversion of IPP to β-Amyrin (Table 2). The expression level of unigenes encoding key enzymes involved in the triterpenoid biosynthesis pathways is shown in the heat map. Most of the unigenes encoding 4-diphosphocytidyl-2-C-methyl-D-erythritol kinase (CMK) and HDR showed the highest expression in leaves, while most of the unigenes encoding acetyl-CoA acetyltransferase (AACT), hydroxymethylglutaryl-CoA synthase (HMGS), HMGR, diphosphomevalonate decarboxylase (PMD), SE, and β-AS showed the highest expression in roots. Unigenes encoding DXR and 4-hydroxy-3-methylbut-2-enyl diphosphate synthase (HDS) were highly expressed in either roots or leaves (Fig. 5).

**Table 2:** Distribution of triterpenoid saponin biosynthesis unigenes in four *C. gracile* tissues

Abbreviation	EC	Unigene number	No.in flower	No.in leaf	No.in stem	No.in root
AACT	2.3.1.9	48	18	13	20	32
HMGS	2.3.3.10	21	7	3	10	17
HMGR	1.1.1.34	17	8	10	9	16
MK	2.7.1.36	5	2	2	2	5
PMK	2.7.4.2	5	5	5	5	5
PMD	4.1.1.33	12	7	6	7	10
DXS	2.2.1.7	36	18	18	17	23
DXR	1.1.1.267	2	1	1	1	2
MCT	2.7.7.60	3	3	3	3	3
CMK	2.7.1.148	5	3	2	3	4
MDS	4.6.1.12	3	2	2	2	3
HDS	1.17.7.1	2	2	2	2	2
HDR	1.17.7.4	11	7	9	7	8
IDI	5.3.3.2	12	7	6	7	11
GPPS	2.5.1.1	7	4	4	4	5
FPPS	2.5.1.10	27	18	18	16	19
SS	2.5.1.21	19	13	13	13	16
SE	1.14.14.17	14	6	5	6	12
β-AS	5.4.99.39	32	26	18	22	27

**Fig. 6:**
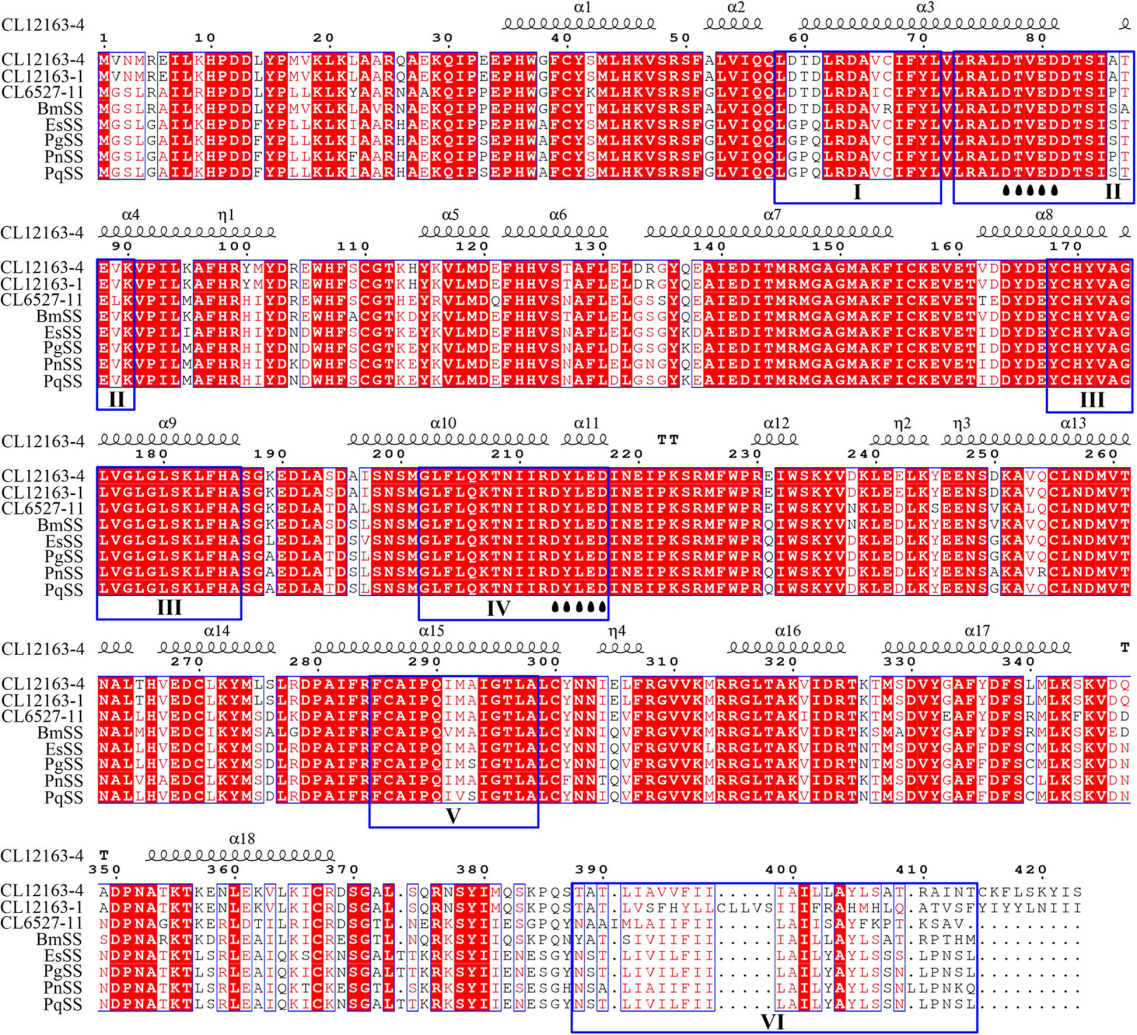
Sequence alignment and secondary structure of CgSS. Amino acid sequences of SS enzymes were retrieved from GenBank. BmSS, *Bacopa monniera* (ADX01171.1); EsSS, *Eleutherococcus senticosus* (AEA41712.1); PgSS, *Panax ginseng* (ACV88718.1); PnSS, *Panax notoginseng* (ABA29019.1); and PqSS, *Panax quinquefolius* (CAJ58418.1). The six domains in SS protein are outlined in blue. DXXXD domains are indicated using ‘’. White letters on a red background represent identical amino acids, red letters on a white background represent similar amino acids, and black letters represent different amino acids

Three unigenes (CL12163–1, CL12163–4, CL6527–11) encoding the SS enzyme were identified, with a sequence identity of 88.14%. We analyzed the structure of the CL12163–4 unigene, as it showed the highest expression in leaves. The CgSS (*C. gracile* squalene synthase) ORF with 1272 bp is expected to encode a protein of 423 amino acids. The amino acid sequence of CgSS showed high sequence similarity to SS proteins from other herbs, namely, *Bacopa monniera* (ADX01171.1; 83.02%), *Eleutherococcus senticosus* (AEA41712.1; 77.91%), *Panax ginseng* (ACV88718.1; 78.14%), *Panax notoginseng* (ABA29019.1; 77.91%), and *Panax quinquefolius* (CAJ58418.1; 77.91%). In the multiple sequence alignment of these SS amino acid sequences, six domains (I–VI) and two aspartate-rich regions, important for the catalytic activity of SS enzymes, are highlighted. Two domains (III and IV) of SS amino acid sequences were highly conserved among plant species, three domains (I, II, and V) were fairly well conserved, whereas domain VI was the least conserved. The secondary structure of CgSS contained 18 alpha helixes, which were the main component of the SS enzyme (Fig. 6).

**Fig. 7:**
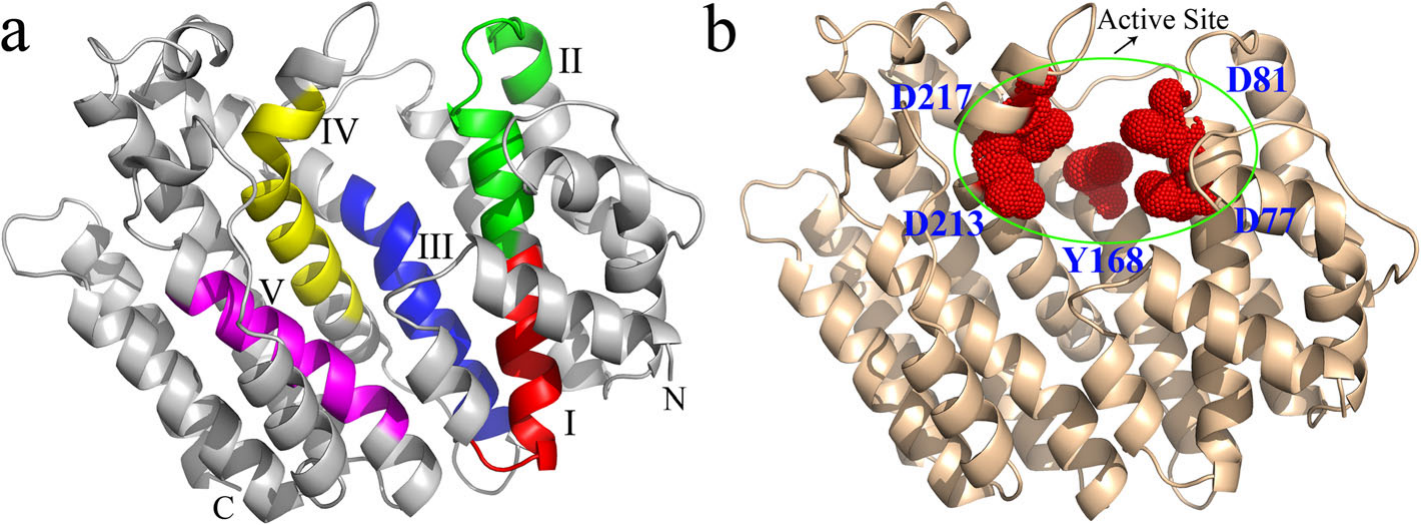
Tertiary structure model and schematic diagram of the CgSS active site. **a** Cartoon display of the three-dimensional structure of CgSS and five conserved domains (I: red, II: green, III: blue, IV: yellow, V: magenta). **b** Schematic diagram showing the active site of CgSS (green ellipse) and five amino acid residues (D77, D81, Y168, D213, and D217; red dots).) The figures were performed using the Swiss Model (https://www.swissmodel.expasy.org) and the PyMOL software based on the the crystal structure of *Trypanosoma cruzi* SS (Template 3wca.3.A, with sequence identity of 46.02%, the ranges from E34–S370)

The 3D model of CgSS was constructed on the basis of the crystal structure of SS from *Trypanosoma cruzi* (PDB ID 3wca.3.A), which shares 46.02% sequence identity with CgSS [27]. Five domains (I–V) of CgSS are highlighted in different colors in Fig. 7a, and amino acids D77, D81, Y168, D213, and D217 comprising the active site of CgSS are indicated in Fig. 7b.

### Validation of RNA-Seq data by qRT-PCR

**Fig. 8:**
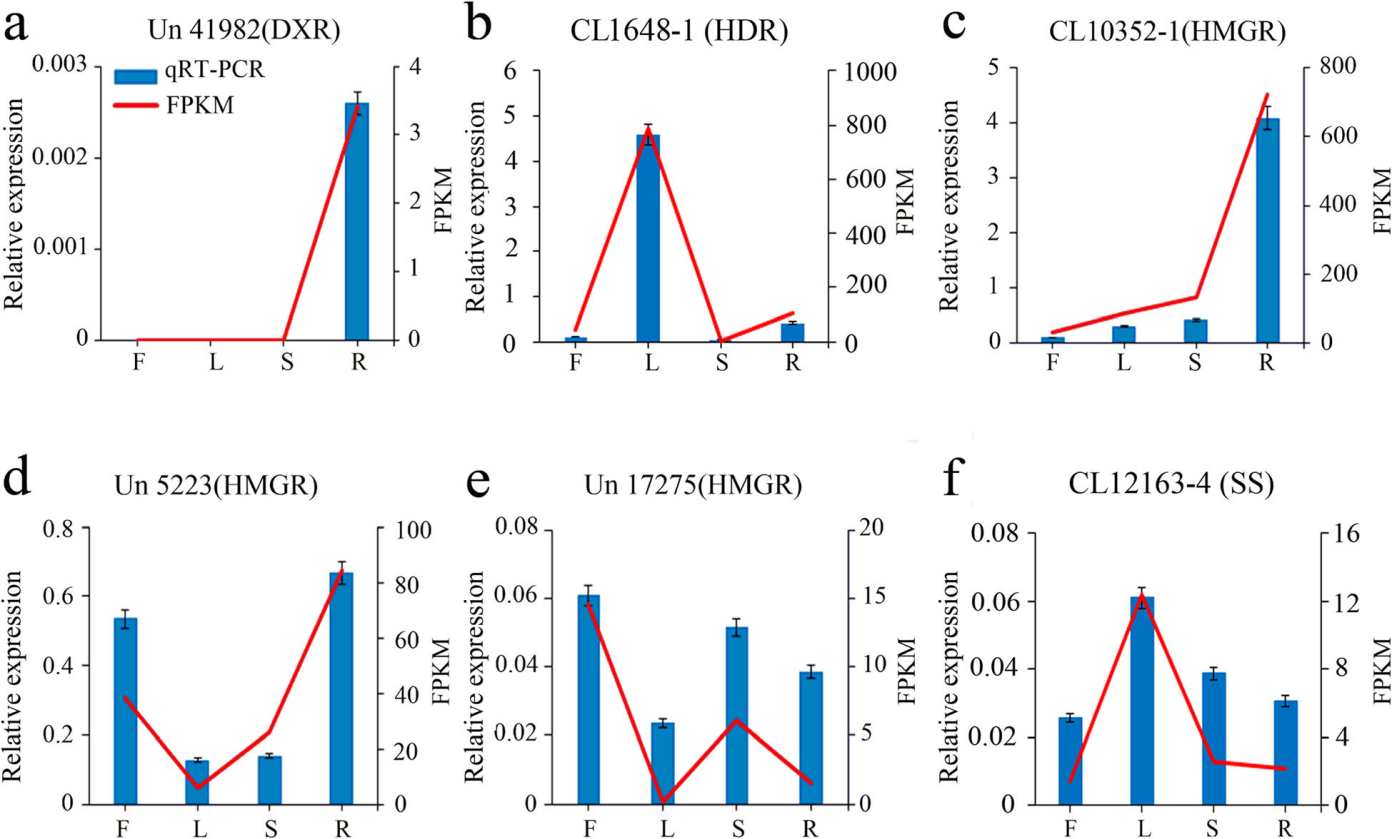
Quantitative real-time PCR (qRT-PCR) analysis of six unigenes encoding enzymes involved in the triterpenoid biosynthesis pathway in *C. gracile. ***a** Un 41,982 encoding DXR. **b** CL1648-1 encoding HDR. **c** CL10352–1 encoding HMGR. **d** Un 5223 encoding HMGR. **e** Un 17, 275 encoding HMGR. **f** CL12163–4 encoding SS. Blue bars show the results of the qRT-PCR analysis, and red lines show the FPKM values determined by RNA-Seq analysis. The left Y-axis indicates the relative expression level of the qRT-PCR detection gene, and the right Y-axis indicates the FPKM value in the RNA-Seq data. F, flowers; L, leaves; S, stems; R, roots. Data represent mean ± standard error (SE; *n* = 3)

To independently verify the transcriptome data and differential gene expression among different tissues, six unigenes involved in triterpenoid saponin biosynthesis were selected for qRT-PCR analysis. The expression levels of Un 41,982 (DXR), CL10352–1 (HMGR), and Un 5223 (HMGR) were the highest in roots. Similarly, the expression levels of CL12163–4 (SS), and CL1648–1 (HDR) were the highest in leaves, while that of Un 17,275 (HMGR) was the highest in flowers. These data were consistent with the FPKM values of these genes determined from RNA-Seq analysis (Fig. 8).

### Identification of DEGs

**Fig. 9:**
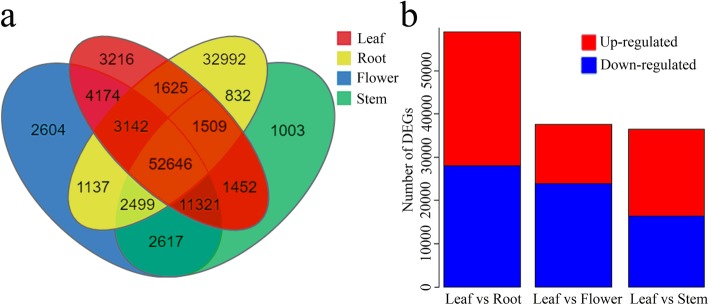
Expression of unigenes in all four tissues of *C. gracile*. **a** The number of unigenes expressed in four tissues by veen diagram. **b** Number of DEGs identified in the leaves compared with roots, flowers, and stems of *C. gracile*. Genes highly expressed in leaves compared with roots, flowers, and stems were defined as “up-regulated,” whereas genes with lower expression levels in leaves were defined as “down-regulated”

A total of 52,646 unigenes were co-expressed in all four tissues, whereas 3216 unigenes were expressed only in leaves (Fig. 9a). DEGs were identified based on the comparison of expression profiles of unigenes between leaves and the other tissues (flowers, stems, and roots) (Fig. 9b). A comparison of gene expression between leaves and flowers revealed 37,744 DEGs, of which 13,899 were up-regulated and 23,845 were down-regulated in leaves. Comparison of gene expression between leaves and roots revealed 59,154 DEGs, of which 31,166 were up-regulated and 27,988 were down-regulated in leaves. Comparison of gene expression between leaves and stems revealed 36,467 DEGs, of which 20,104 were up-regulated and 16,363 were down-regulated in leaves. A total of 239, 397, and 332 unigenes were up-regulated in leaves compared with flowers, roots, and stems, respectively (Table 3).

**Table 3:** KEGG pathway annotations of terpenoid metabolic genes up-regulated in leaves compared with other tissues

Terpenoids metabolic Pathway	Pathway ID	Number of up-regulated genes		
		Leaf vs Flower	Leaf vs Root	Leaf vs Stem
Terpenoid backbone biosynthesis	ko00900	47	89	74
Monoterpenoid biosynthesis	ko00902	39	32	32
Diterpenoid biosynthesis	ko00904	21	53	43
Carotenoid biosynthesis	ko00906	61	97	79
Sesquiterpenoid and triterpenoid biosynthesis	ko00909	25	47	44
Ubiquinone and other terpenoid-quinone biosynthesis	ko00130	46	79	60

**Fig. 10:**
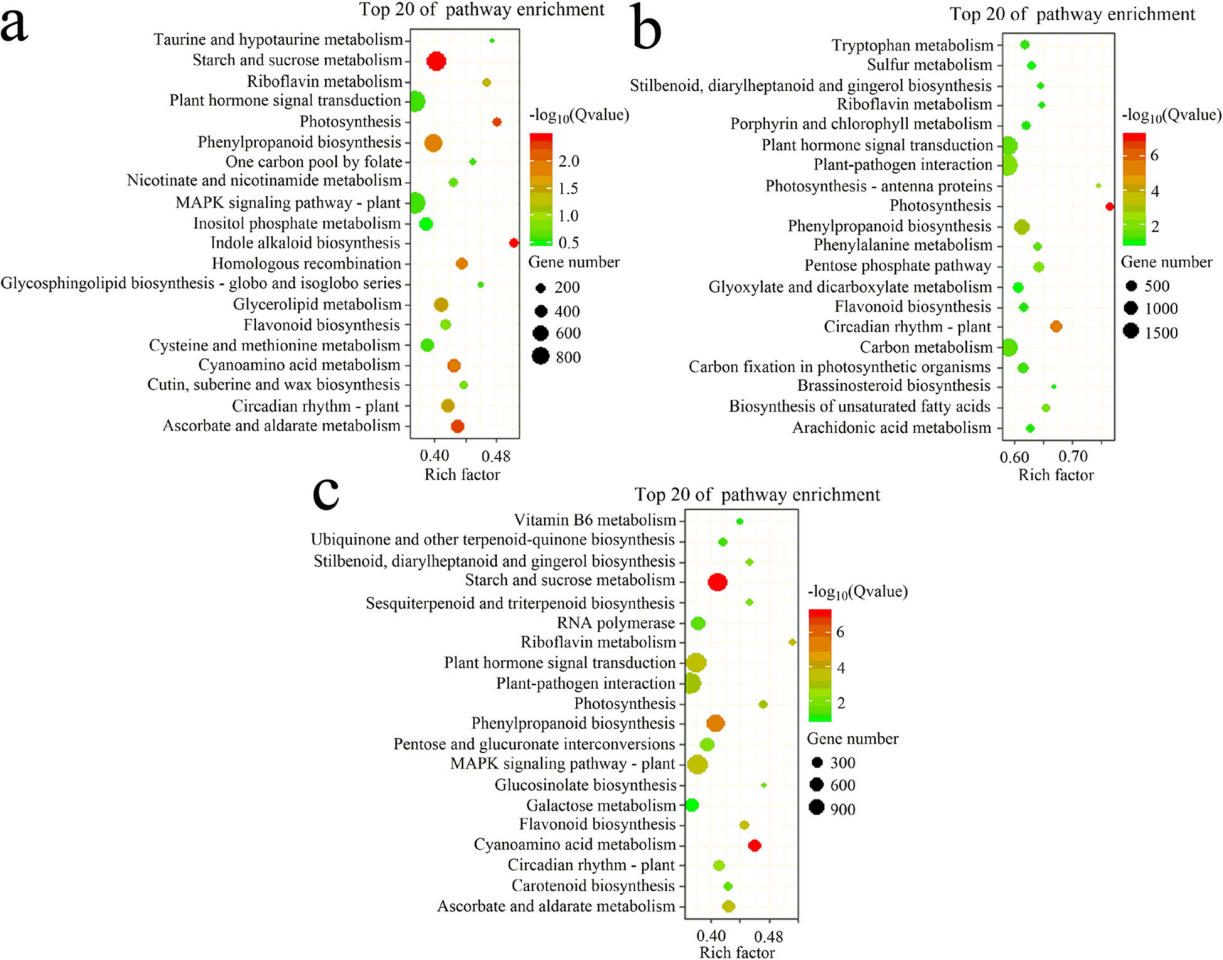
The DEGs significantly enriched in KEGG pathways. (**a**–**c**) Significantly enriched pathways in the DEGs identified in the comparison between leaves vs. flowers (**a**), leaves vs. roots (**b**) and leaves vs. stems (**c**)

The 37,744 DEGs identified in the leaves vs. flowers comparison were mainly enriched in “Indole alkaloid biosynthesis,” “Photosynthesis,” “Taurine and hypotaurine metabolism,” “Riboflavin metabolism,” and “Glycosphingolipid biosynthesis globo and isoglobo series” (Fig. 10a). The 59,154 DEGs identified in the leaves vs. roots comparison were primarily enriched in “Photosynthesis,” “Circadian rhythm plant,” “Photosynthesis antenna proteins,” “Brassinosteroid biosynthesis,” and “Biosynthesis of unsaturated fatty acids” (Fig. 10b). The 36,467 DEGs identified in the leaves vs. stems comparison were primarily enriched in “Riboflavin metabolism,” “Glucosinolate biosynthesis,” “Photosynthesis,” “Cyanoamino acid metabolism,” and “Sesquiterpenoid and triterpenoid biosynthesis” (Fig. 10c).

### Identification of leaf-specific expression unigenes

**Fig. 11:**
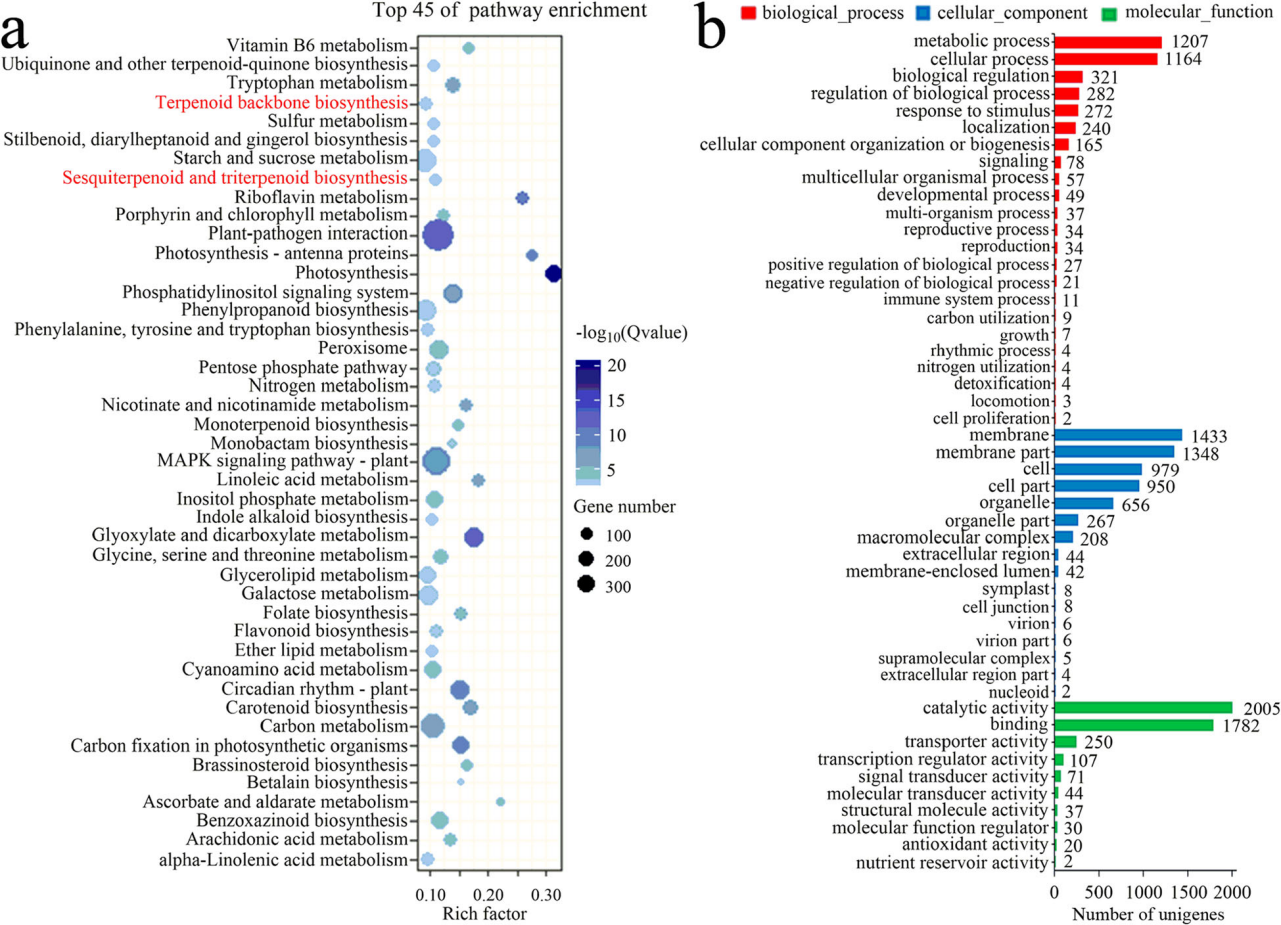
KEGG and GO annotations of unigenes expressed specifically in the leaves of *C. gracile*. **a** Significantly enriched pathways among unigenes showing leaf-specific expression. The triterpenoid saponin biosynthesis pathways are indicated in red. **b** Classification of leaf-specific unigenes annotated using the GO database

A total of 9094 up-regulated genes showing leaf-specific expression (log_2_FC > 1) were mapped to 132 pathways in the KEGG database; these DEGs were mainly enriched in “Photosynthesis,” “Photosynthesis-antenna proteins,” “Riboflavin metabolism,” “Benzoxazinoid biosynthesis,” and “Linoleic acid metabolism” (Fig. 11a). In the GO database, unigenes with rhizome-specific expression were mapped to 49 subcategories in three functional categories, with the highest enrichment in “metabolic process,” “cellular process,” and “biological regulation” under “biological process”; “membrane,” “membrane part,” and “cell” under “cellular component”; and “catalytic activity,” “binding,” and “transporter” activity under “molecular function” (Fig. 11b).

### Analysis of genes encoding TFs related to the biosynthesis of terpenoids and other secondary metabolites

**Fig. 12:**
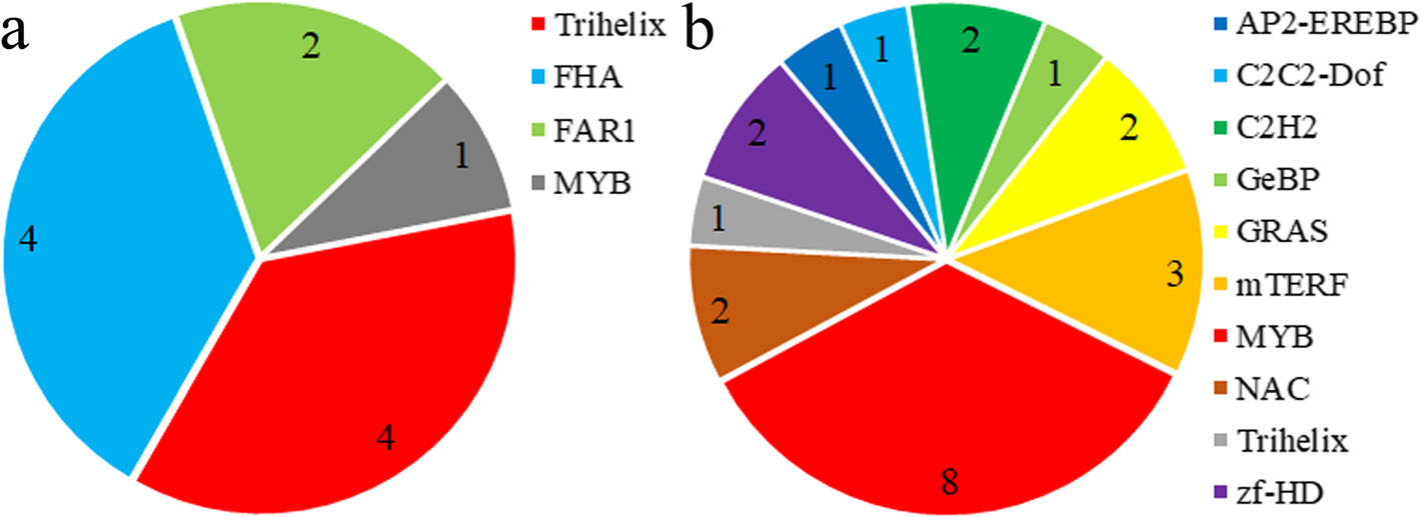
The number of unigenes encoding TFs involved in the metabolic pathways in *C. gracile*. **a** TF families and unigene numbers related to the metabolism of terpenoids. **b** TF families and unigene numbers related to “metabolism of biosynthesis of other secondary metabolites”

Transcription factor (TF) families play key roles in regulating the activity of genes involved in triterpenoid saponin biosynthesis and other secondary metabolic processes by specifically binding to cis-regulatory elements in their promoter regions. A total of 3536 unigenes encoding TFs were identified in the *C. gracile* transcriptome. These included 517, 1128, and 742 TF-encoding unigenes that were up-regulated in leaves compared with flowers, roots, and stems, respectively (Table 4). Most of these unigenes encoded TFs belonging to the MYB (404 unigenes), AP2-EREBP (268 unigenes), bHLH (273 unigenes), WRKY (200 unigenes), C3H (175 unigenes), and NAC (156 unigenes) families. Moreover, unigenes encoding FHA (4 unigenes), Trihelix (4 unigenes), FAR1 (2 unigenes), and MYB (1 unigenes) TFs were involved in terpenoid metabolism. A total of 23 unigenes encoding TF participated in the biosynthesis of secondary metabolites (Additional file 8: Table S4, Fig. 12a and b).

**Table 4:** Number of transcription factor (TF) genes showing differential expression in different tissues of *C. gracile*

TF family	Number of unigenes	Number of up-regulated unigenes		
		Leaf vs Flower	Leaf vs Root	Leaf vs Stem
MYB	404	78	144	87
bHLH	273	46	99	68
AP2-EREBP	268	35	55	33
WRKY	200	65	71	62
C3H	175	18	53	27
NAC	156	37	42	40
GRAS	113	18	26	20
ARF	113	6	40	9
G2-like	106	17	40	28
C2H2	106	12	30	16
MADS	101	10	31	22
ABI3VP1	99	6	25	12
FAR1	95	11	47	24
mTERF	88	9	42	21
Trihelix	75	4	20	11
C2C2-GATA	75	7	19	15
Tify	68	8	18	9
HSF	63	8	11	7
C2C2-Dof	63	11	15	14
SBP	59	6	31	10
Alfin-like	54	3	19	16
FHA	52	10	14	11
TAZ	50	4	15	5
RWP-RK	50	14	17	23
GeBP	20	2	7	7
zf-HD	13	1	7	3
Other	597	71	190	142
Total number	3536	517	1128	742

## Discussion

Triterpenoid saponins are the major bioactive compounds in *C. gracile*, with extensive pharmacological effects. However, genomic information on saponin biosynthesis is limited. In this study, we aimed to identify putative genes involved in triterpenoid saponin biosynthesis in *C. gracile*. Therefore, we conducted deep transcriptome sequencing on different tissues of *C. gracile* plants using RNA-Seq. Analysis of RNA-Seq data revealed 128,856 unigenes, with a mean length of 1354 bp and an N50 value of 2268 bp. Of the 128,856 unigenes, 99,020 were annotated, whereas 23.15% of the unigenes remained unannotated, probably due to the lack of public data of plant transcriptome and genome. The assembly quality of *C. gracile* transcriptome was better than that of other medicinal plants in the Labiatae family such as Red *Perilla frutescens var. crispa* (no. of unigenes = 47,788; mean unigene length = 876 bp; N50 = 1349 bp) [28], *Salvia guaranitica* L. (no. of unigenes = 61,400; mean unigene length = 731 bp; N50 = 1334 bp) [29], and *Salvia miltiorrhiza* (no. of unigenes = 50,778; mean unigene length = 868.75 bp; N50 = 1618 bp) [30]. These results demonstrate the reliability of the *C. gracile* transcriptome.

We performed transcriptome analysis of the flowers, leaves, roots, and stems of *C. gracile* to facilitate the dissection of genes involved in the tissue-specific biosynthesis of triterpenoid saponins. This approach is widely used for the identification of novel genes involved in the biosynthesis of secondary metabolites and analysis of the molecular mechanism of triterpenoid saponin biosynthesis [31].

In the transcriptome data sets of *C. gracile*, the up-regulated unigenes in leaves were mainly enriched in “Terpenoid backbone biosynthesis” and “Sesquiterpenoid and triterpenoid biosynthesis” in the KEGG database and were mainly annotated under “metabolic process” and “catalytic activity” in the GO database. These up-regulated genes may explain the molecular basis of the medicinal value of *C. gracile* leaves.

The sponin content of different *C. gracile* tissues was determined by UV-spectrophotometry, with buddlejasaponin IV as a standard, which was identified and confirmed by UPLC/Q-TOF-MS. Saponins include steroid and triterpenoid saponins in most of plant species. Triterpenoid saponins are generally predominant in dicotyledons, while steroidal saponins are mainly identified in monocotyledons [32]. Triterpenoid saponins are some of the most important components of *C. gracile*, although the relationship between the expression of triterpenoid biosynthesis genes and accumulation of triterpenoid saponins in *C. gracile* is unclear.

In this study, UPLC/Q-TOF-MS analysis was performed to identify the different constituents of triterpenoid saponins. UV-spectrophotometry analysis confirmed that the content of total saponin in *C. gracile* was the highest in leaves compared with other tissues. Higher expression levels of some of the unigenes encoding SS, HDR, HDS, DXS, and DXR enzymes in leaves compared with stems, flowers, and roots were consistent with the higher saponin accumulation in *C. gracile* leaves (Fig. 1 and Fig. 5). This suggests that overexpression of genes encoding these key enzymes could increase the production of triterpenoid saponins in *C. gracile* plants. A similar approach, i.e., overexpression of the *SS* gene has been previously used to enhance the production of total saponins in *Medicago truncatula* [33].

Roots showed the highest expression of unigenes encoding most of the key enzymes involved in the biosynthesis of triterpenoid saponins (Fig. 5); however, this result contradicts the high levels of triterpenoid saponins in leaves. Expression levels of genes do not always correlate with enzyme activity [34]. Post-transcriptional modifications influence features of mRNAs, thus affecting protein synthesis. For example, in a previous study, the mismatch between ginsenoside content and HMGR gene transcript levels was reportedly affected by post-transcriptional modifications and feedback regulation [35]. Post-translational modifications such as phosphorylation and ubiquitination can also determine the rate of protein degradation [36].

The expression levels of CL10352–1 (HMGR), Un 5223 (HMGR), Un 17,275 (HMGR), Un 41,982 (DXR), CL12163–4 (SS), and CL1648–1 (HDR) determined by qRT-PCR were consistent to the FPKM values determined by RNA-Seq, thus confirming the reliability of transcriptomic data, which will be helpful for understanding the biosynthesis pathway of triterpenoid saponins in *C. gracile*.

Multiple sequence alignment of SS amino acid sequences suggested the presence of six domains (I–VI) required for the functional activity of the enzyme. Domains I, II, and III are involved in the first step of SS biosynthesis; domain IV participates in the second step; domain VI is a hydrophobic region that binds to the endoplasmic reticulum. The Mg^2+^-binding aspartate-rich regions and amino acids in the active site (Asp77, Asp81, Tyr168, Asp213, and Asp217) were highly conserved in SS amino acid sequences among different plant species [37–39]. Previously, it has been shown that overexpression of the *SS* (AB115496) gene significantly increases the ginsenoside content [40] in *Panax ginseng*. Therefore, our findings will be beneficial for further investigation into the role of SS in triterpenoid saponin biosynthesis.

TFs are sequence-specific DNA-binding proteins that modulate target gene expression. In the *C. gracile* transcriptome, only one unigene (Un 15,683) encoding an MYB TF was annotated to play a role in the metabolism of terpenoids. Previous studies have shown that the overexpression of *MYB9b* enhances tanshinone concentration in *Salvia miltiorrhiza* hairy roots [41], and MYB1 increases artemisinin content and trichome proliferation in *Artemisia annua* [42]. Additionally, eight unigenes encoding MYB TFs were mapped to “metabolism of biosynthesis of other secondary metabolites” in *C. gracile*. Overexpression of the gene encoding MYB1 and MYB2 TFs enhances the synthesis and accumulation of anthocyanins in *Fagopyrum tataricum* [43]. Moreover, MYB5, MYB26, and MYB31 participate in flavonoid biosynthesis in *Ginkgo biloba* under adverse conditions [44]. These TFs may also play a significant role in the regulation of triterpenoid saponins and other secondary metabolites in *C. gracile*.

## Conclusion

Overall, this is the first report of a comprehensive RNA-Seq analysis of *C. gracile* tissues to identify genes involved in the biosynthesis of triterpenoid saponins. The transcriptome data will facilitate further examination of the molecular mechanism of triterpenoid saponin biosynthesis in *C. gracile* and promote the study of *C. gracile* functional genomics.

## Methods

### Plant material and RNA isolation

Ten plants of *C. gracile* (height = ~ 18 cm) were harvested from the herbal garden of the Anhui University of Chinese Medicine under the permission of managers and professionals on April 18, 2018 (Additional file 9: Figure S5), which were identified by Prof. Qingshan Yang (Anhui University of Chinese Medicine). Flower, leaf, root, and stem tissues were separated from three *C. gracile* plants, cleaned with ultrapure water, dried on filter paper, and then frozen in liquid nitrogen.

Total RNA was isolated from four tissues (flowers, leaves, roots, and stems) of *C. gracile* plants in three replicates using the ethanol precipitation protocol and RNA Plant Kit (Aidlab Biotech, Beijing, China), according to the manufacturer’s instructions. The quality and quantity of total RNA were verified using NanoDrop 8000 and Agilent 2100 Bioanalyzer (Thermo Fisher Scientific, MA, USA), respectively (Additional file 10: Table S5).

### Determination of total saponin content

Saponins were extracted from dried *C. gracile* tissues (flowers, leaves, roots, and stems) and detected, as described previously [45], with slight modifications. Briefly, dried powder (0.1 g) of each tissue was extracted in 2 ml of 50% methanol by ultrasonication for 30 min. The filtrate obtained by vacuum filtration was concentrated at 60 °C to near dryness and then resuspended in 25 ml of 50% methanol. The absorbance of the solution was determined by UV-spectrophotometry (JASCO Company, Japan). A curve of concentration versus absorbance was established using buddlejasaponin IV, as a standard, to determine the saponin content of *C. gracile* tissue (Additional file 11: Figure S6a and b). Total saponin yield (%) was calculated as follows:

$$ \mathrm{Yield}\ \left(\%\right)=\left[\left(\frac{A+0.0081}{0.0741}\right)\times \left(\frac{1}{40m}\right)\right]\times 100\%. $$

where A and m represent the solution absorbance of sample and weight of sample dried powder, respectively.

### UPLC/Q-TOF-MS analysis

Triterpenoid saponin metabolite profiling was performed on a Waters ACQUITY UPLC /Xevo G2 Q-TOF (Waters Corporation, Milford, MA, USA). Samples were separated on Agilent ZORBAX Eclipse Plus C18 (2.1 mm × 50 mm, 1.8 μm). The column temperature was maintained at 35 °C, and the flow rate was 0.20 mL/min. The mobile phase consisted of acetonitrile and 0.1% formic acid. The gradient was initiated with 10% acetonitrile for 5 min, 10% acetonitrile linearly increased to 20% acetonitrile within 1 min and held at 20% for 4 min, then 20% acetonitrile increased to 30% acetonitrile within 1 min and held at 30% for 4 min, and 30% acetonitrile increased to 45% acetonitrile within 1 min and held at 45% for 4 min. The Xevo G2-S Q-TOF mass spectrometer was run in the negative mode. Mass spectra were obtained with a scanning mass range of 50 to 1500 Da. High-purity nitrogen (N_2_) was used as nebulizing gas, and ultra-high pure helium (He) was used as the collision gas. Other parameters were as follows: Capillary voltage, 2.00 kV; sampling cone voltage, 40.0 V; ion source temperature, 120 °C; desolvation temperature, 350 °C; cone gas flow, 50 L/h; desolvation gas flow rate, 600 L/h; collision energy (CE), 40–60 V; and leucine enkephalin was used as lock mass (m/z 554.2615).

### cDNA library preparation and RNA-Seq analysis

Total RNA isolated from plant tissues was treated with RNase-free DNase I (TaKaRa, China) to eliminate any traces of contaminating DNA, which was then mixed with oligo (dT) magnetic beads to purify mRNA. The purified mRNA was fragmented at an appropriate temperature. First-strand cDNA was generated by reverse transcription PCR (RT-PCR) using random hexamer primers. This was followed by second-strand cDNA synthesis. Subsequently, A-Tailing Mix (Enzymatics, USA) and RNA Index Adapters were added to perform end-repair, and the resulting cDNA was amplified by PCR. The PCR products were purified by Ampure XP Beads and eluted in EB solution. The quantity and quality of cDNA libraries were evaluated using Agilent 2100 Bioanalyzer (ABI, New York, NY, USA). The double-stranded PCR products were heated denatured and circularized by the splint oligo sequence to obtain the final library. The single-stranded circular DNA was amplified using phi29 (Thermo Scientific, USA) to generate DNA nanoball (DNB). The DNBs were loaded into the patterned nanoarray and single-end 50 bp reads were generated on the BGISEQ-500 platform (Beijing Genomics Institute, Wuhan, China) [46].

### De novo transcriptome assembly

To obtain high-quality reads, low quality reads (i.e., reads with > 20% nucleotides with base quality < 10) and reads with adaptor sequences and/or unknown nucleotides (> 5%) were filtered using SOAPnuke [47] and Trimmomatic. De novo assembly of the high-quality reads was performed using Trinity [48]; PCR duplicates were removed prior to assembly to improve assembly efficiency. The assembled transcripts were clustered using the TGI clustering tool (TGICL) [49] to remove redundant sequences and obtain non-redundant sequences, termed unigenes.

### Annotation of unigenes

The functional annotation of unigenes was performed by searching for homologous sequences in public databases, including NT (NCBI nucleotide; ftp://ftp.ncbi.nlm.nih.gov/blast/db), NR (NCBI non-redundant protein sequence; ftp://ftp.ncbi.nlm.nih.gov/blast/db), KOG (clusters of euKaryotic Orthologous Groups; http://www.ncbi.nlm.nih.gov/KOG), KEGG (Kyoto Encyclopedia of Genes and Genome; http://www.genome.jp/kegg), GO (Gene Ontology; http://geneontology.org), Pfam (protein families; http://pfam.xfam.org), and SwissProt (a manually annotated and reviewed protein sequence database; http://ftp.ebi.ac.uk/pub/databases/swissprot). Sequences in the NT database were searched using Blastn [50], while those in the NR, KOG, SwissProt, and KEGG databases were searched using Blastx [51]. With NR annotation, the Blast2GO (version 2.5.0) was used to obtain the GO annotations of unigenes [52], while unigenes were mapped to the Pfam database using Hmmscan.

The full-length open reading frame (ORF) of *C. gracile SS* (*CgSS*) was translated using the ExPASy translation tool (https://web.expasy.org/translate/), and multiple sequence alignment was implemented using DNAMAN 6.0.3.99 and Clustalx 1.83 software. Conserved domains in the amino acid sequence of CgSS were detected using the Conserved Domains Database (http://www.ncbi.nlm.nih.gov/Structure/cdd/wrpsb.cgi/). The secondary structure of CgSS was predicted using ESPript 2.2 (http://espript.ibcp.fr/ESPript/cgi-bin/ESPript.cgi). Three-dimensional (3D) homologous modeling of CgSS was performed using the Swiss Model (https://www.swissmodel.expasy.org), and the 3D structure was described using the PyMOL software.

### Differentially expressed genes analysis

High-quality reads of each sample were queried against the genome sequence of *C. gracile* using Bowtie2 (version 2.2.5) [53], and gene expression level in each sample was calculated using RSEM (version 1.2.8) [54]. Unigenes showing significant differences in expression levels (fold-change [FC] ≥ 2.00; false discovery rate [FDR] ≤ 0.001) between leaves and other tissues (root, flowers, and stems) were identified by the PoissonDis method [55–57]. These DEGs were then used for GO functional analysis and KEGG pathways enrichment.

### Analysis of transcription factor (TF) encoding genes

The ORF of unigenes was identified by Getorf (EMBOSS:6.5.7.0) [58], and then annotated to the TF domains in the plant transcription factor database (PlantTFDB) using Hmmsearch (version 3.0) [59].

### Analysis of the key genes expression level in triterpenoid saponin biosynthesis by qRT-PCR

To validate the *C. gracile* transcriptome data sets, qRT-PCR was performed in triplicate using GoTaq qPCR Master Mix (Promega) on a Real-time Thermal Cycler 5100 System (Thermo Scientific, Waltham, MA, USA). Gene-specific primers for the actin gene (Un 11,691) and six unigenes (CL10352–1, Un 17,275, Un 41,982, Un 5223, CL12163–4, and CL1648–1) involved in triterpenoid biosynthesis were designed using Primer Premier (version 5.0) (Additional file 12: Table S6). Each PCR reaction was prepared in a 15-μl mixture volume containing diluted cDNA (2 μl), forward primer (1 μl), reverse primer (1 μl), qPCR Mixture (2X, 7.5 μl), and RNase-free water (3.5 μl) using the following conditions: initial denaturation at 95 °C for 2 min, followed by 40 cycles of denaturation at 95 °C for 15 s, and annealing at 60 °C for 30 s. The relative expression level of each selected unigene was normalized with the CgActin gene (Un 11,691) and detected by the 2^-ΔΔCt^ method [60].

## Supplementary information

**Additional file 1: Table S1.** Total saponin content of leaves, stems, flowers, and roots of *Clinopodium gracile* (Benth.) Matsum.

**Additional file 2: Table S2.** Identification of the constituents of triterpenoid saponins in *C.gracile* by UPLC/Q-TOF-MS.

**Additional file 3: Figure S1.** Length distribution of *C. gracile* unigenes.

**Additional file 4: Figure S2.** Percent homology between the sequences of *C. gracile* and other plant species determined using the NR database.

**Additional file 5: Figure S3.** GO function annotation of *C. gracile* transcriptome.

**Additional file 6: Figure S4.** KEGG functional classification of the annotated unigenes in *C. gracile*.

**Additional file 7: Table S3.** KEGG annotations of all unigenes.

**Additional file 8: Table S4.** Number of unigenes encoding TFs involved in terpenoid metabolism.

**Additional file 9: Figure S5.** Photograph of *C. gracile* plants. The picture was photographed for the plants of *C. gracile* in the herbal garden of the Anhui University of Chinese Medicine on April 18, 2018.

**Additional file 10: Table S5.** Characteristics of RNA isolated from different tissues of *C. gracile*.

**Additional file 11: Figure S6.** (a) The ultraviolet Absorption Spectrum of the buddlejasaponin IV. (b) Standard curve of buddlejasaponin IV at 250 nm.

**Additional file 12: Table S6.** List of genes amplified using the indicated primers by qRT-PCR.

## Data Availability

RNA-Seq data sets of *C. gracile* have been deposited in the NCBI Sequence Read Archive (SRA) database under the accession number SRP194041.

## Abbreviations

AACT: Acetyl-CoA acetyltransferase
*C. gracile*: *Clinopodium gracile* (Benth.) Matsum
CgSS: *C. gracile* squalene synthase
CMK: 4-diphosphocytidyl-2-C-methyl-D-erythritol kinase
CYP450: Cytochrome P450 monooxygenase
DEGs: Differentially expressed genes
DXR: 1-deoxy-D-xylulose-5-phosphate reductase
DXS: 1-deoxy-D-xylulose-5-phosphate synthase
FC: Fold-change
FDR: False discovery rate
FPP: Farnesyl pyrophosphate
FPPS: Farnesyl diphosphate synthase
GO: Gene ontology
GPP: Geranyl pyrophosphate
GPPS: Geranyl diphosphate synthase
HDR: 4-hydroxy-3-methylbut-2-en-1-yl diphosphate reductase
HDS: 4-hydroxy-3-methylbut-2-enyl diphosphate synthase
HMGR: 3-hydroxy-3-methylglutaryl coenzyme A reductase
HMGS: Hydroxymethylglutaryl-CoA synthase
IDI: Isopentenyl-diphosphate delta-isomerase
IPP: Isopentenyl pyrophosphate
KEGG: Kyoto encyclopedia of genes and genome
MCT: 2-C-methyl-D-erythritol 4-phosphate cytidylyltransferase
MDS: 2-C-methyl-D-erythritol 2,4-cyclodiphosphate synthase
MEP: The 2-C-methyl-D-erythrirtol-4-phosphate
MK: Mevalonate kinase
MVA: The mevalonic acid
NGS: Next-generation sequencing
NR: NCBI non-redundant protein sequence
ORF: Open reading frame
PlantTFDB: The plant transcription factor database
PMD: Diphosphomevalonate decarboxylase
PMK: Phosphomevalonate kinase
PSPP: Presqualene diphosphate
qRT-PCR: Quantitative real-time PCR
RNA-Seq: RNA sequencing
SE: Squalene epoxidase
SS: Squalene synthase
TFs: Transcription factors
UGT: UDP-glycosyltransferase
β-AS: Beta-amyrin synthase
