# Supplementary material for: Transcriptome analysis of Clinopodium gracile (Benth.) Matsum and identification of genes related to Triterpenoid Saponin biosynthesis
Source: BMC Genomics. 2020 Jan 15;21:49. doi: 10.1186/s12864-020-6454-y (PMC6964110; doi:10.1186/s12864-020-6454-y)
Supplement: Supplementary file 1 — Additional file 1: Table S1. Total saponin content of leaves, stems, flowers, and roots of Clinopodium gracile (Benth.) Matsum. [file 12864_2020_6454_MOESM1_ESM.docx]

**Additional file 1: Table S1.** Total saponin content of leaves, stems, flowers and roots of *Clinopodium gracile* (Benth.) Matsum.

| Samples | | Dried powder (g) | Absorbance | Content (mg) | Percentage (%) | Mean Percentage (%) | Standard error |
| --- | --- | --- | --- | --- | --- | --- | --- |
| Leaf | 1 | 0.1010 | 0.865 | 0.2946 | 0.2917 | 0.2920 | 0.0005 |
|  | 2 | 0.1010 | 0.863 | 0.2939 | 0.2910 |  |  |
|  | 3 | 0.1000 | 0.861 | 0.2932 | 0.2932 |  |  |
| Stem | 1 | 0.1053 | 0.705 | 0.2406 | 0.2285 | 0.2286 | 0.0004 |
|  | 2 | 0.1050 | 0.701 | 0.2392 | 0.2278 |  |  |
|  | 3 | 0.1030 | 0.693 | 0.2365 | 0.2296 |  |  |
| Flower | 1 | 0.1110 | 0.657 | 0.2244 | 0.2022 | 0.2059 | 0.0033 |
|  | 2 | 0.1090 | 0.643 | 0.2197 | 0.2016 |  |  |
|  | 3 | 0.1020 | 0.639 | 0.2183 | 0.2140 |  |  |
| Root | 1 | 0.0700 | 0.372 | 0.1282 | 0.1831 | 0.1815 | 0.0011 |
|  | 2 | 0.07080 | 0.375 | 0.1293 | 0.1826 |  |  |
|  | 3 | 0.07020 | 0.364 | 0.1255 | 0.1788 |  |  |
