# Supplementary material for: Transcriptome analysis of Clinopodium gracile (Benth.) Matsum and identification of genes related to Triterpenoid Saponin biosynthesis
Source: BMC Genomics. 2020 Jan 15;21:49. doi: 10.1186/s12864-020-6454-y (PMC6964110; doi:10.1186/s12864-020-6454-y)
Supplement: Supplementary file 2 — Additional file 2: Table S2. Identification of the constituents of triterpenoid saponins in C.gracile by UPLC/Q-TOF-MS. [file 12864_2020_6454_MOESM2_ESM.docx]

**Additional file 2: Table S2.** Identification of the constituents of triterpenoid saponins in *C.gracile* by UPLC/Q-TOF-MS.

| NO | Formula | Identification | t_R_(min) | Measured (m/z) | Calculated (m/z) | Maior fragment ions (m/z) | MS Error (ppm) |
| --- | --- | --- | --- | --- | --- | --- | --- |
| 1 | C_42_H_68_O_13_ | Saikosaponin a | 23.357 | 779.4645 | 779.4582 | 779.4645[M-H]^-^, 617.4078[M-H-Glc]^-^ | 8.08 |
| 2 | C_48_H_78_O_18_ | Buddlejasaponin IV | 21.236 | 941.5182 | 941.5110 | 941.5182[M-H]^-^, 779.4645[M-H-Glc]^-^, 617.4078[M-H-Glc- Glc]^-^ | 7.65 |
| 3 | C_54_H_88_O_23_ | Clinoposaponin III | 19.629 | 1103.5720 | 1103.5638 | 1103.5720 [M-H]^-^,  957.5153[M-H-Rha]^-^, 811.4886[M-H-Rha-Rha]^-^,649.4319[M-H-Rha-Rha-Glc]^-^ | 7.43 |
| 4 | C_60_H_98_O_28_ | Clinoposaponin Ⅴ | 15.023 | 1265.6277 | 1265.6166 | 1265.6277[M-H]^-^,  1103.5789[M-H-Glc]^-^,959.5299[M-H-Rha]^-^,  797.4779[M-H-Rha-Glc]^-^,635.4147[M-H-Rha-Glc-Glc]^-^ | 8.77 |
