# Supplementary figures and images for: Transcriptome analysis of Clinopodium gracile (Benth.) Matsum and identification of genes related to Triterpenoid Saponin biosynthesis
Source: BMC Genomics. 2020 Jan 15;21:49. doi: 10.1186/s12864-020-6454-y (PMC6964110; doi:10.1186/s12864-020-6454-y)

**
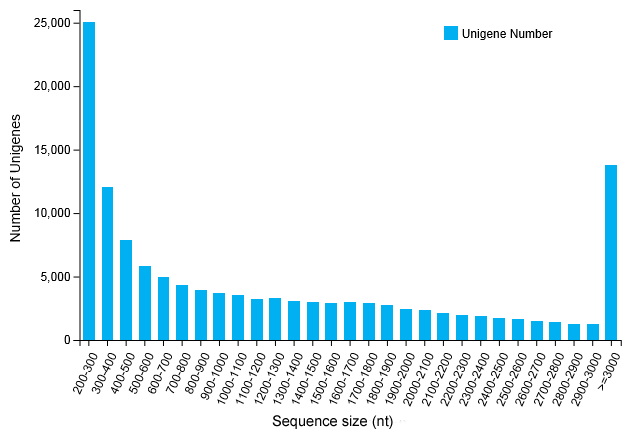
**

**Additional file 3: Figure S1.** Length distribution of *C. gracile* unigenes.

Supplement: Supplementary file 3 — Additional file 3: Figure S1. Length distribution of C. gracile unigenes. [file 12864_2020_6454_MOESM3_ESM.docx]

**
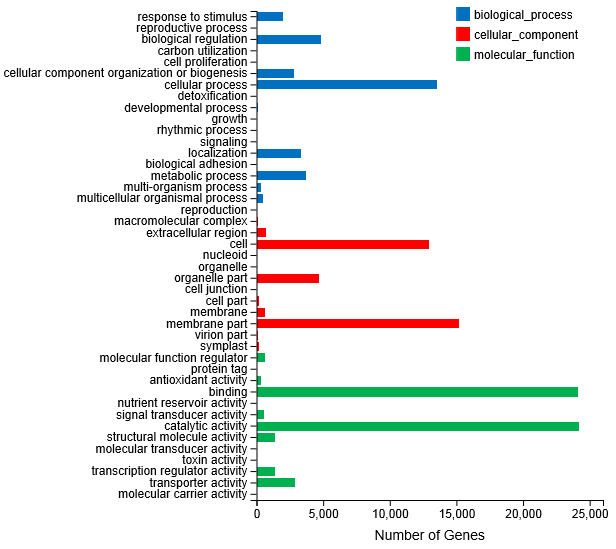
** **Additional file 5: Figure S3.** GO function annotation of *C. gracile* transcriptome.

Supplement: Supplementary file 5 — Additional file 5: Figure S3. GO function annotation of C. gracile transcriptome. [file 12864_2020_6454_MOESM5_ESM.docx]
