# Supplementary material for: Transcriptome analysis of Clinopodium gracile (Benth.) Matsum and identification of genes related to Triterpenoid Saponin biosynthesis
Source: BMC Genomics. 2020 Jan 15;21:49. doi: 10.1186/s12864-020-6454-y (PMC6964110; doi:10.1186/s12864-020-6454-y)
Supplement: Supplementary file 4 — Additional file 4: Figure S2. Percent homology between the sequences of C. gracile and other plant species determined using the NR database. [file 12864_2020_6454_MOESM4_ESM.docx]

**
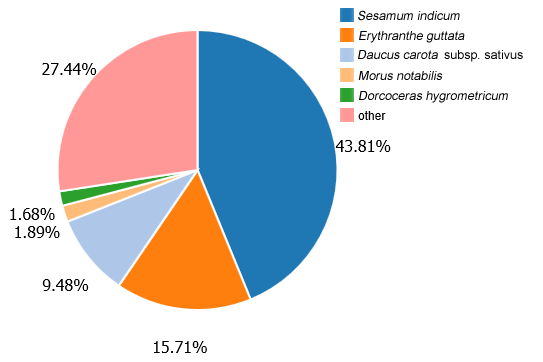
**

**Additional file 4: Figure S2.** Percent homology between the sequences of *C. gracile* and other plant species determined using the NR database.
