# Supplementary material for: Transcriptome analysis of Clinopodium gracile (Benth.) Matsum and identification of genes related to Triterpenoid Saponin biosynthesis
Source: BMC Genomics. 2020 Jan 15;21:49. doi: 10.1186/s12864-020-6454-y (PMC6964110; doi:10.1186/s12864-020-6454-y)
Supplement: Supplementary file 6 — Additional file 6: Figure S4. KEGG functional classification of the annotated unigenes in C. gracile. [file 12864_2020_6454_MOESM6_ESM.docx]

**
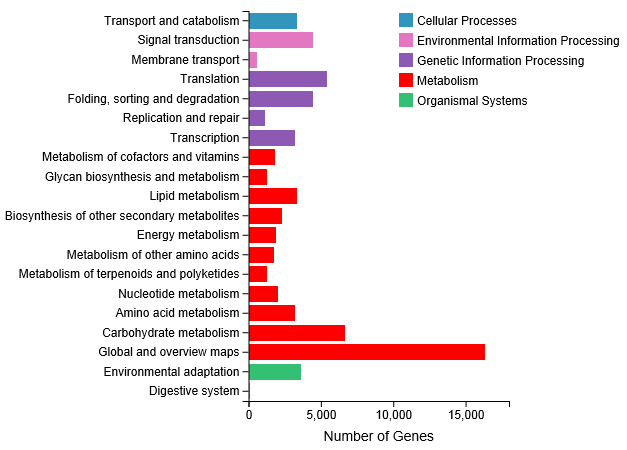
**

**Additional file 6: Figure S4.** KEGG functional classification of the annotated unigenes in *C. gracile*.
