# Supplementary material for: Transcriptome analysis of Clinopodium gracile (Benth.) Matsum and identification of genes related to Triterpenoid Saponin biosynthesis
Source: BMC Genomics. 2020 Jan 15;21:49. doi: 10.1186/s12864-020-6454-y (PMC6964110; doi:10.1186/s12864-020-6454-y)
Supplement: Supplementary file 7 — Additional file 7: Table S3. KEGG annotations of all unigenes. [file 12864_2020_6454_MOESM7_ESM.docx]

**Additional file 7: Table S3.** KEGG annotations of all unigenes.

| Pathway Name | All genes with pathway annotation (73648) | Pathway ID |
| --- | --- | --- |
| Metabolic pathways | 15329 | ko01100 |
| Biosynthesis of secondary metabolites | 8082 | ko01110 |
| Plant-pathogen interaction | 3098 | ko04626 |
| MAPK signaling pathway – plant | 2285 | ko04016 |
| Plant hormone signal transduction | 2209 | ko04075 |
| RNA transport | 2079 | ko03013 |
| Spliceosome | 1945 | ko03040 |
| Carbon metabolism | 1828 | ko01200 |
| Endocytosis | 1741 | ko04144 |
| Protein processing in endoplasmic reticulum | 1740 | ko04141 |
| Biosynthesis of amino acids | 1686 | ko01230 |
| Purine metabolism | 1682 | ko00230 |
| Ribosome | 1681 | ko03010 |
| Starch and sucrose metabolism | 1673 | ko00500 |
| Pyrimidine metabolism | 1488 | ko00240 |
| Phenylpropanoid biosynthesis | 1406 | ko00940 |
| mRNA surveillance pathway | 1275 | ko03015 |
| Ubiquitin mediated proteolysis | 1152 | ko04120 |
| Amino sugar and nucleotide sugar metabolism | 1093 | ko00520 |
| RNA degradation | 1003 | ko03018 |
| Galactose metabolism | 984 | ko00052 |
| Glycolysis / Gluconeogenesis | 908 | ko00010 |
| RNA polymerase | 904 | ko03020 |
| Pentose and glucuronate interconversions | 818 | ko00040 |
| Peroxisome | 796 | ko04146 |
| Glycerophospholipid metabolism | 749 | ko00564 |
| Glycerolipid metabolism | 745 | ko00561 |
| Phosphatidylinositol signaling system | 731 | ko04070 |
| Pyruvate metabolism | 685 | ko00620 |
| Ribosome biogenesis in eukaryotes | 672 | ko03008 |
| Oxidative phosphorylation | 664 | ko00190 |
| Cysteine and methionine metabolism | 656 | ko00270 |
| Inositol phosphate metabolism | 626 | ko00562 |
| ABC transporters | 614 | ko02010 |
| Cyanoamino acid metabolism | 612 | ko00460 |
| Phagosome | 607 | ko04145 |
| Ascorbate and aldarate metabolism | 598 | ko00053 |
| Fatty acid metabolism | 591 | ko01212 |
| Glutathione metabolism | 569 | ko00480 |
| Circadian rhythm – plant | 556 | ko04712 |
| Sphingolipid metabolism | 556 | ko00600 |
| 2-Oxocarboxylic acid metabolism | 535 | ko01210 |
| Glyoxylate and dicarboxylate metabolism | 508 | ko00630 |
| Carbon fixation in photosynthetic organisms | 507 | ko00710 |
| Aminoacyl-tRNA biosynthesis | 488 | ko00970 |
| Valine, leucine and isoleucine degradation | 482 | ko00280 |
| Fatty acid degradation | 474 | ko00071 |
| Arginine and proline metabolism | 454 | ko00330 |
| Nucleotide excision repair | 443 | ko03420 |
| Fructose and mannose metabolism | 437 | ko00051 |
| Glycine, serine and threonine metabolism | 429 | ko00260 |
| Pentose phosphate pathway | 419 | ko00030 |
| Homologous recombination | 417 | ko03440 |
| Other glycan degradation | 411 | ko00511 |
| Alanine, aspartate and glutamate metabolism | 399 | ko00250 |
| Tryptophan metabolism | 397 | ko00380 |
| N-Glycan biosynthesis | 385 | ko00510 |
| Proteasome | 375 | ko03050 |
| Flavonoid biosynthesis | 367 | ko00941 |
| Citrate cycle (TCA cycle) | 364 | ko00020 |
| DNA replication | 362 | ko03030 |
| Terpenoid backbone biosynthesis | 360 | ko00900 |
| Basal transcription factors | 352 | ko03022 |
| Lysine degradation | 349 | ko00310 |
| Protein export | 346 | ko03060 |
| alpha-Linolenic acid metabolism | 345 | ko00592 |
| beta-Alanine metabolism | 338 | ko00410 |
| Porphyrin and chlorophyll metabolism | 330 | ko00860 |
| Phenylalanine, tyrosine and tryptophan biosynthesis | 322 | ko00400 |
| Tyrosine metabolism | 315 | ko00350 |
| Glycosaminoglycan degradation | 305 | ko00531 |
| Propanoate metabolism | 304 | ko00640 |
| Base excision repair | 296 | ko03410 |
| Phenylalanine metabolism | 289 | ko00360 |
| Arginine biosynthesis | 281 | ko00220 |
| Autophagy - other | 279 | ko04136 |
| Nitrogen metabolism | 273 | ko00910 |
| Pantothenate and CoA biosynthesis | 273 | ko00770 |
| Biosynthesis of unsaturated fatty acids | 272 | ko01040 |
| Carotenoid biosynthesis | 269 | ko00906 |
| Mismatch repair | 264 | ko03430 |
| Ether lipid metabolism | 264 | ko00565 |
| Arachidonic acid metabolism | 258 | ko00590 |
| Ubiquinone and other terpenoid-quinone biosynthesis | 248 | ko00130 |
| Fatty acid biosynthesis | 248 | ko00061 |
| Glycosphingolipid biosynthesis - ganglio series | 232 | ko00604 |
| Nicotinate and nicotinamide metabolism | 228 | ko00760 |
| Butanoate metabolism | 227 | ko00650 |
| Sulfur metabolism | 216 | ko00920 |
| Folate biosynthesis | 214 | ko00790 |
| Glycosylphosphatidylinositol (GPI)-anchor biosynthesis | 212 | ko00563 |
| Photosynthesis | 212 | ko00195 |
| SNARE interactions in vesicular transport | 203 | ko04130 |
| Selenocompound metabolism | 203 | ko00450 |
| Diterpenoid biosynthesis | 198 | ko00904 |
| Indole alkaloid biosynthesis | 187 | ko00901 |
| Stilbenoid, diarylheptanoid and gingerol biosynthesis | 181 | ko00945 |
| Isoquinoline alkaloid biosynthesis | 177 | ko00950 |
| Cutin, suberine and wax biosynthesis | 176 | ko00073 |
| Steroid biosynthesis | 173 | ko00100 |
| Linoleic acid metabolism | 169 | ko00591 |
| Valine, leucine and isoleucine biosynthesis | 165 | ko00290 |
| Sesquiterpenoid and triterpenoid biosynthesis | 163 | ko00909 |
| Fatty acid elongation | 160 | ko00062 |
| Riboflavin metabolism | 154 | ko00740 |
| Histidine metabolism | 145 | ko00340 |
| Tropane, piperidine and pyridine alkaloid biosynthesis | 142 | ko00960 |
| Other types of O-glycan biosynthesis | 135 | ko00514 |
| Vitamin B6 metabolism | 125 | ko00750 |
| Thiamine metabolism | 123 | ko00730 |
| Biotin metabolism | 119 | ko00780 |
| Zeatin biosynthesis | 115 | ko00908 |
| Monoterpenoid biosynthesis | 115 | ko00902 |
| Glucosinolate biosynthesis | 110 | ko00966 |
| One carbon pool by folate | 109 | ko00670 |
| Synthesis and degradation of ketone bodies | 105 | ko00072 |
| Brassinosteroid biosynthesis | 103 | ko00905 |
| Photosynthesis – antenna proteins | 98 | ko00196 |
| Cholesterol metabolism | 93 | ko04979 |
| Monobactam biosynthesis | 93 | ko00261 |
| Lysine biosynthesis | 91 | ko00300 |
| Isoflavonoid biosynthesis | 89 | ko00943 |
| Glycosphingolipid biosynthesis - globo and isoglobo series | 85 | ko00603 |
| C5-Branched dibasic acid metabolism | 78 | ko00660 |
| Phosphonate and phosphinate metabolism | 73 | ko00440 |
| Anthocyanin biosynthesis | 72 | ko00942 |
| Non-homologous end-joining | 60 | ko03450 |
| Flavone and flavonol biosynthesis | 60 | ko00944 |
| Taurine and hypotaurine metabolism | 59 | ko00430 |
| Sulfur relay system | 53 | ko04122 |
| Benzoxazinoid biosynthesis | 36 | ko00402 |
| Betalain biosynthesis | 26 | ko00965 |
| Lipoic acid metabolism | 22 | ko00785 |
| Caffeine metabolism | 16 | ko00232 |
| Mannose type O-glycan biosynthesis | 12 | ko00515 |
| Glycosphingolipid biosynthesis – lacto and neolacto series | 7 | ko00601 |
