# Supplementary material for: Transcriptome analysis of Clinopodium gracile (Benth.) Matsum and identification of genes related to Triterpenoid Saponin biosynthesis
Source: BMC Genomics. 2020 Jan 15;21:49. doi: 10.1186/s12864-020-6454-y (PMC6964110; doi:10.1186/s12864-020-6454-y)
Supplement: Supplementary file 8 — Additional file 8: Table S4. Number of unigenes encoding TFs involved in terpenoid metabolism. [file 12864_2020_6454_MOESM8_ESM.docx]

**Additional file 8: Table S4.** Number of unigenes encoding TFs involved in terpenoid metabolism.

| TF family | Number of unigenes | Pathway ID | Pathway name |
| --- | --- | --- | --- |
| Trihelix | 4 | ko00130 | Ubiquinone and other terpenoid-quinone biosynthesis |
| FHA | 4 | ko00906 | Carotenoid biosynthesis |
| FAR1 | 2 | ko00906 | Carotenoid biosynthesis |
| MYB | 1 | ko00906 | Carotenoid biosynthesis |
