# Supplementary material for: Transcriptome analysis of Clinopodium gracile (Benth.) Matsum and identification of genes related to Triterpenoid Saponin biosynthesis
Source: BMC Genomics. 2020 Jan 15;21:49. doi: 10.1186/s12864-020-6454-y (PMC6964110; doi:10.1186/s12864-020-6454-y)
Supplement: Supplementary file 9 — Additional file 9: Figure S5. Photograph of C. gracile plants. The picture was photographed for the plants of C. gracile in the herbal garden of the Anhui University of Chinese Medicine on April 18, 2018. [file 12864_2020_6454_MOESM9_ESM.docx]

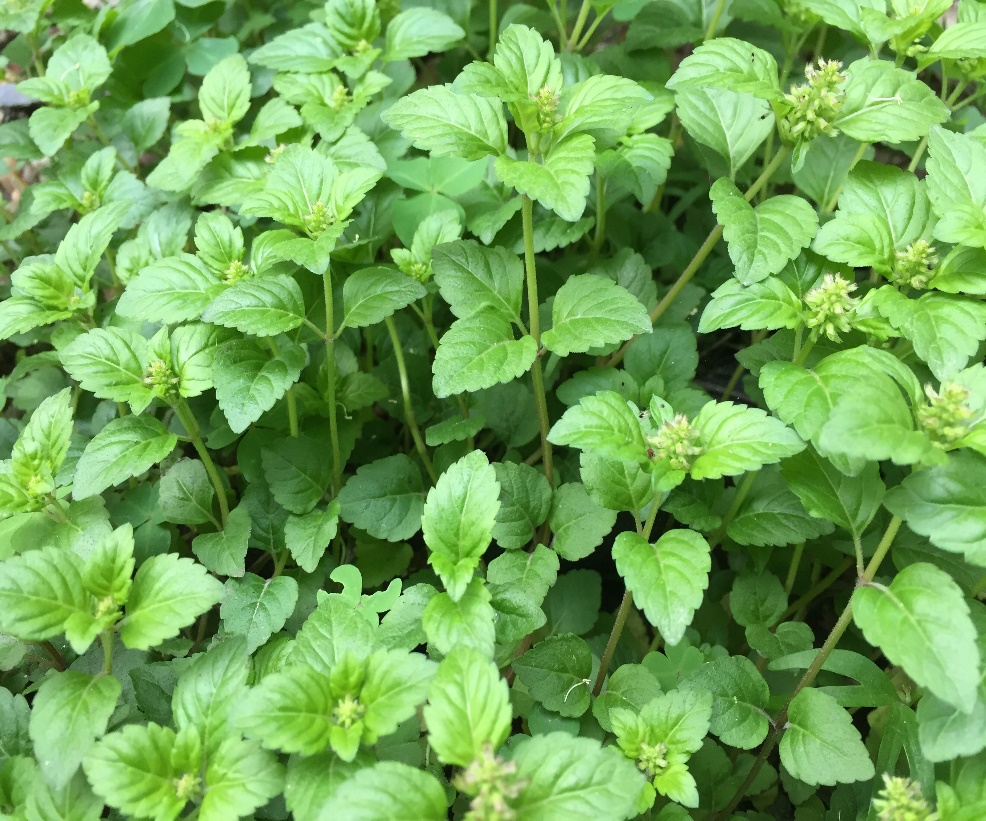


**Additional file 9: Figure S5.** Photograph of *C. gracile* plants. The picture was photographed for the plants of *C. gracile* in the herbal garden of the Anhui University of Chinese Medicine on April 18, 2018.
