# Supplementary material for: Transcriptome analysis of Clinopodium gracile (Benth.) Matsum and identification of genes related to Triterpenoid Saponin biosynthesis
Source: BMC Genomics. 2020 Jan 15;21:49. doi: 10.1186/s12864-020-6454-y (PMC6964110; doi:10.1186/s12864-020-6454-y)
Supplement: Supplementary file 10 — Additional file 10: Table S5. Characteristics of RNA isolated from different tissues of C. gracile. [file 12864_2020_6454_MOESM10_ESM.docx]

**Additional file 10: Table S5.** Characteristics of RNA isolated from different tissues of *C.gracile*.

| Sample | Concentration (ng/μL) | Total amount (μg) | OD260/280 | RIN | 28S/18S |
| --- | --- | --- | --- | --- | --- |
| Leaves | 164 | 13.12 | 1.97 | 7.2 | 1.6 |
| Roots | 738 | 14.76 | 1.94 | 8.6 | 1.6 |
| Flowers | 696 | 55.68 | 1.83 | 9.2 | 2.0 |
| Stems | 165 | 8.25 | 1.97 | 9.0 | 1.6 |
