# Supplementary material for: Transcriptome analysis of Clinopodium gracile (Benth.) Matsum and identification of genes related to Triterpenoid Saponin biosynthesis
Source: BMC Genomics. 2020 Jan 15;21:49. doi: 10.1186/s12864-020-6454-y (PMC6964110; doi:10.1186/s12864-020-6454-y)
Supplement: Supplementary file 11 — Additional file 11: Figure S6. (a) The ultraviolet Absorption Spectrum of the buddlejasaponin IV. (b) Standard curve of buddlejasaponin IV at 250 nm. [file 12864_2020_6454_MOESM11_ESM.docx]

**
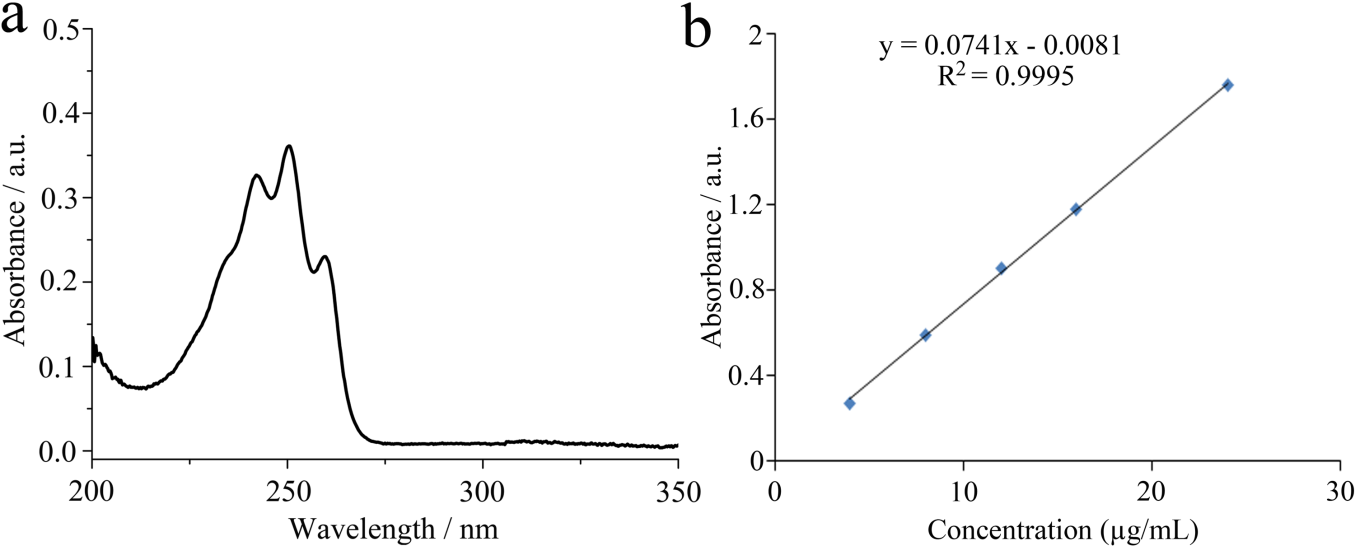
**

**Additional file 11: Figure S6.** (a) The ultraviolet Absorption Spectrum of the buddlejasaponin IV. (b) Standard curve of buddlejasaponin IV at 250 nm.
