# Supplementary material for: Transcriptome analysis of Clinopodium gracile (Benth.) Matsum and identification of genes related to Triterpenoid Saponin biosynthesis
Source: BMC Genomics. 2020 Jan 15;21:49. doi: 10.1186/s12864-020-6454-y (PMC6964110; doi:10.1186/s12864-020-6454-y)
Supplement: Supplementary file 12 — Additional file 12: Table S6. List of genes amplified using the indicated primers by qRT-PCR. [file 12864_2020_6454_MOESM12_ESM.docx]

**Additional file 12: Table S6.** List of genes amplified using the indicated primers by qRT-PCR.

| Genes | Amplicon Size （bp） | Primer pairs | TM value |
| --- | --- | --- | --- |
| *actin* | 116 | 5'- TCGATGATCGGAATGGAAGC-3' | 56.7 |
|  |  | 5'- CACCACTGAGAACAATGTTG -3' | 56.5 |
| Un 41982 | 110 | 5'- AGGCACTATGACTGGAGTTC -3' | 56.4 |
|  |  | 5'- CACAAGTTAGCTCCACAACC -3' | 56.6 |
| CL10352-1 | 103 | 5'- CACTGCATCACGATGATGGA -3' | 56.8 |
|  |  | 5'- ATTGAGTCCCACCTCCTACT -3' | 56.7 |
| Un 5223 | 171 | 5'- ATGGACGTCCTTGGCATCTC -3' | 56.9 |
|  |  | 5'- GAGCATGTTGAGTTCCACCA -3' | 56.5 |
| Un 17275 | 105 | 5'-ACTGGTCAAGATCCAGCTCA -3' | 56.3 |
|  |  | 5'-GTACCAACCTCGATAGATGG -3' | 56.6 |
| CL12163-4 | 287 | 5'- CTCGCGAAATTTGGAGTAAA -3' | 56.1 |
|  |  | 5'- AAGCCAGCTATTGAAATCGC -3' | 56.1 |
| CL1648-1 | 224 | 5'- CCCGGATAAGGATCTTGAGA -3' | 56.7 |
|  |  | 5'- TGGTCGTACTCTAGGTGGAA -3' | 56.5 |
